# Supplementary material for: Investigating the impact of enhanced community case management and monthly screening and treatment on the transmissibility of malaria infections in Burkina Faso: study protocol for a cluster-randomised trial
Source: BMJ Open. 2019 Sep 13;9(9):e030598. doi: 10.1136/bmjopen-2019-030598 (PMC6747640; doi:10.1136/bmjopen-2019-030598)
Supplement: Supplementary data [file bmjopen-2019-030598supp001.pdf]

Supplementary Information

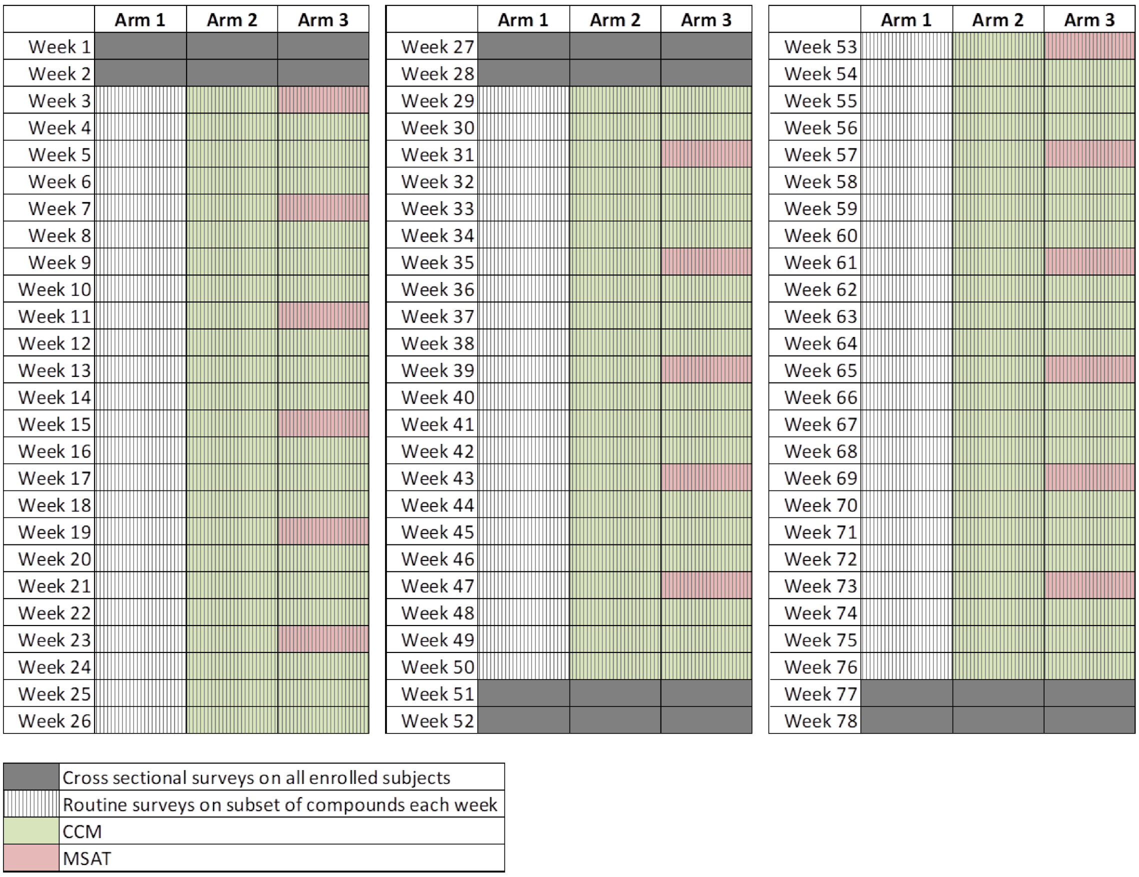

Figure S1. Timetable of trial activities
CCM = enhanced community case management; MSAT = monthly screening and treatment

Collins KA, *et al. BMJ Open* 2019; 9:e030598. doi: 10.1136/bmjopen-2019-030598

Data category	Information
**Primary registry and trial identifying number**	ClinicalTrials.gov: NCT03705624
**Date of registration in primary registry**	July 19, 2018
**Secondary identifying numbers**	INDIE-1a
**Source(s) of monetary or material support**	The Bill and Melinda Gates Foundation
**Primary sponsor**	London School of Hygiene and Tropical Medicine<br>Keppel Street<br>London WC1E 7HT<br>Tel: +44 207 927 2626<br>Email: RGIO@lshtm.ac.uk
**Secondary sponsor(s)**	N/A
**Contact for public queries**	Teun Bousema, PhD +31 0243617574 (teun.bousema@radboudumc.nl)<br>Katharine Collins, PhD +31 0243613663 (katharine.collins@radboudumc.nl)<br>Chris Drakeley, PhD +44 02079272289 (chris.drakeley@lshtm.ac.uk)<br>Alfred Tiono, MD, PhD +226 50324695 (t.alfred@fasonet.bf)
**Contact for scientific queries**	TeunBousema, PhD +31 243617574 (teun.bousema@radboudumc.nl)<br>Chris Drakeley, PhD +44 2079272289 (chris.drakeley@lshtm.ac.uk)<br>Alfred Tiono, MD, PhD +226 50324695 (t.alfred@fasonet.bf)<br>Katharine Collins, PhD+31 243613663 (katharine.collins@radboudumc.nl)
**Public title**	P. falciparum Infection Dynamics and Transmission to Inform Elimination (INDIE 1a)
**Scientific title**	A cluster-randomized trial investigating the impact of enhanced community case management and monthly screening and treatment on the transmissibility of malaria infections in Burkina Faso
**Countries of recruitment**	Burkina Faso
**Health condition(s) or problem(s) studied**	Malaria
**Intervention(s)**	<u>Arm 1 (control): Standard of care</u><br>• Standard of care with passively monitored malaria incidence<br><u>Arm 2 (intervention): CCM</u><br>• Standard of care plus enhanced Community Case Management for Malaria (CCM) involving weekly active screening for fever. A measured temperature ≥37.5°C or reported fever in last 24h will prompt screening with a conventional rapid diagnostic test (RDT). RDT positive individuals will be treated with artemether-lumefantrine according to national guidelines<br><u>Arm 3 (intervention): CCM + MSAT</u><br>• Standard of care with enhanced CCM plus monthly screening and treatment regardless of symptoms using a conventional RDT (MSAT). RDT positive individuals will be treated with artemether-lumefantrine according to national guidelines.
**Key inclusion and exclusion criteria**	<u>Inclusion Criteria:</u><br>1. Participants should be permanent residents of the compound<br>2. Participants should be willing to participate in repeated assessments of health and infection status and willing to donate a maximum of 37mL of blood (children <10 years of age) or 52mL of blood (older individuals) during an 18-month period<br><br><u>Exclusion Criteria:</u><br>1. Any (chronic) illness that would affect with study participation<br>2. Pre-existing severe chronic health conditions

2

	3. Current participation in malaria vaccine trials or participation in such trials in the last 2 years<br>4. History of intolerance to artemether-lumefantrine
**Study type**	Interventional<br>Allocation: randomized<br>Intervention model: Cluster randomized trial<br>Masking: none (open label)<br>Primary purpose: Diagnostic
**Date of first enrolment**	June 2018
**Target sample size**	900
**Recruitment status**	Recruiting
**Primary outcome(s)**	Parasite prevalence and density between arms by molecular detection at the end of study cross-sectional survey [Time Frame: Month 18 (end of second transmission season; January-February 2020)].
**Key secondary outcomes**	1. Parasite prevalence and density by molecular detection at the end of year 1 cross-sectional survey [Time Frame: Month 6 (end of first transmission season; January-February 2019)].<br>2. Parasite prevalence and density by molecular detection at the end of the dry season cross-sectional survey [Time Frame: Month 12 (prior to second transmission season; June 2019)].<br>3. Gametocyte prevalence and or density by molecular methods at the end of study cross-sectional survey [Time Frame: Month 18 (end of second transmission season; January-February 2020)].<br>4. Gametocyte prevalence and or density by molecular methods at the end of year 1 cross-sectional survey [Time Frame: Month 6 (end of first transmission season; January-February 2019)].<br>5. Gametocyte prevalence and or density by molecular methods at the end of the dry season cross-sectional [Time Frame: Month 12 (prior to second transmission season; June 2019)].<br>6. Gametocyte prevalence and or density by molecular methods among *P. falciparum* infections during all visits in the study [Time Frame: Throughout study, an average of 18 months].<br>7. The number of incident infections/clinical incidence detected during CCM, MSAT and passive case detection [Time Frame: Throughout study, an average of 18 months].<br>8. Infectivity to mosquitoes of *P. falciparum* infections [Time Frame: Throughout study, an average of 18 months].

Table S1. World Health Organization Trial Registration Data Set

Collins KA, *et al. BMJ Open* 2019; 9:e030598. doi: 10.1136/bmjopen-2019-030598
