# Supplementary material for: Investigating the impact of enhanced community case management and monthly screening and treatment on the transmissibility of malaria infections in Burkina Faso: study protocol for a cluster-randomised trial
Source: BMJ Open. 2019 Sep 13;9(9):e030598. doi: 10.1136/bmjopen-2019-030598 (PMC6747640; doi:10.1136/bmjopen-2019-030598)
Supplement: Author's manuscript [file bmjopen-2019-030598.draft_revisions.pdf]

BMJ Open

BMJ Open is committed to open peer review. As part of this commitment we make the peer review history of every article we publish publicly available.

When an article is published we post the peer reviewers' comments and the authors' responses online. We also post the versions of the paper that were used during peer review. These are the versions that the peer review comments apply to.

The versions of the paper that follow are the versions that were submitted during the peer review process. They are not the versions of record or the final published versions. They should not be cited or distributed as the published version of this manuscript.

BMJ Open is an open access journal and the full, final, typeset and author-corrected version of record of the manuscript is available on our site with no access controls, subscription charges or pay-per-view fees (http://bmjopen.bmj.com).

If you have any questions on BMJ Open's open peer review process please email info.bmjopen@bmj.com

BMJ Open

Study protocol for a cluster-randomized trial investigating the impact of enhanced community case management and monthly screening and treatment on the transmissibility of malaria infections in Burkina Faso

Journal:	*BMJ Open*
Manuscript ID	bmjopen-2019-030598
Article Type:	Protocol
Date Submitted by the Author:	21-Mar-2019
Complete List of Authors:	Collins, Katharine; Radboudumc, Department or Medical Microbiology<br>Ouedraogo, Alphonse; Centre National de Recherche et de Formation sur le Paludisme<br>Guelbeogo, Wamdaogo ; Centre National de Recherche et de Formation sur le Paludisme<br>Awandu, Shehu; Radboudumc, Department or Medical Microbiology<br>Stone, Will; London School of Hygiene and Tropical Medicine, Department of Immunology and Infection<br>Soulama, Issiaka; Centre National de Recherche et de Formation sur le Paludisme<br>Ouattara, Maurice ; Centre National de Recherche et de Formation sur le Paludisme<br>Nombre, Apollinaire ; Centre National de Recherche et de Formation sur le Paludisme<br>Diarra, Amidou; Centre National de Recherche et de Formation sur le Paludisme<br>Bradley, John; London School of Hygiene and Tropical Medicine, MRC Tropical Epidemiology Group<br>Selvaraj, Prashanth; Institute for Disease Modeling<br>Gerardin, Jaline; Institute for Disease Modeling<br>Drakeley, Chris; London School of Hygiene and Tropical Medicine, Department of Immunology and Infection<br>Bousema, Teun; Radboudumc, Department or Medical Microbiology<br>Tiono, Alfred; Centre National de Recherche et de Formation sur le Paludisme
Keywords:	Malaria, Transmission, Gametocyte, Direct membrane feeding assay, asymptomatic infection, Plasmodium falciparum

SCHOLARONE™
Manuscripts

TITLE:

Study protocol for a cluster-randomized trial investigating the impact of enhanced community case management and monthly screening and treatment on the transmissibility of malaria infections in Burkina Faso

AUTHORS:

Katharine A Collins[1*], Alphonse Ouedraogo[2*], Wamdaogo Moussa Guelbeogo[2], Shehu S. Awandu[1], Will Stone[3], Issiaka Soulama[2], Maurice S. Ouattara[2], Apollinaire Nombre[2], Amidou Diarra[2], John Bradley[4], Prashanth Selvaraj[5], Jaline Gerardin[5], Chris Drakeley[3^#], Teun Bousema[1^#], Alfred B. Tiono[2#]

AFFILIATIONS:

1. Department of Medical Microbiology, Radboud university medical center, Nijmegen, The Netherlands

2. Centre National de Recherche et de Formation sur le Paludisme, Ouagadougou , Burkina Faso

3. Department of Immunology and Infection, London School of Hygiene and Tropical Medicine, Faculty of Infectious and Tropical Diseases, London, United Kingdom

4. MRC Tropical Epidemiology Group, London School of Hygiene and Tropical Medicine, London, United Kingdom

5. Institute for Disease Modeling, Bellevue WA United States

[*] These authors contributed equally

[#] These authors contributed equally

[^]Corresponding authors:
Chris Drakeley. Department of Infection & Immunity, London School of Hygiene & Tropical Medicine, London, UK, Tel: +44 (0) 20 7927 2289, Email: chris.drakeley@lshtm.ac.uk

Teun Bousema. Department of Medical Microbiology, Radboud university medical center, Nijmegen, The Netherlands, Tel: +31 243617574, Email: teun.bousema@radboudumc.nl

For peer review only - http://bmjopen.bmj.com/site/about/guidelines.xhtml

ABSTRACT:

Introduction

A large proportion of malaria-infected individuals in endemic areas do not experience symptoms that prompt treatment-seeking. These asymptomatically infected individuals may retain their infections for many months during which sexual-stage parasites (gametocytes) are produced that may be transmissible to mosquitoes. Reductions in malaria transmission could be achieved by detecting and treating these infections early. This study assesses the impact of enhanced community case management and monthly screening and treatment on the prevalence and transmissibility of malaria infections.

Methods and analysis

This cluster-randomized trial will take place in Sapone, an area of intense, highly seasonal malaria in Burkina Faso. In total, 180 compounds will be randomized to 1 of 3 interventions: arm 1 - current standard of care with passively monitored malaria infections; arm 2 - standard of care plus enhanced community case management (CCM), comprising active weekly screening for fever, and detection and treatment of infections in fever positive individuals using conventional rapid diagnostic tests (RDTs); or arm 3 - standard of care and enhanced CCM, plus monthly screening and treatment (MSAT) using RDTs. The study will be conducted over approximately 18 months covering two high transmission seasons and the intervening dry season. The recruitment strategy is such that overall transmission and force of infection should not be effected so the impact of interventions can be continuously evaluated in the context of on-going intense malaria transmission. The main objectives of the study are to determine the impact of enhanced CCM and MSAT on the prevalence and density of parasitemia and gametocytemia and the transmissibility of infections. This will be achieved by molecular detection of infections in all study participants during start and end season cross-sectional surveys and routine sampling of malaria positive individuals to assess their infectiousness to mosquitoes.

Ethics and dissemination

The study has been approved by the relevant institutional ethics committees in Burkina Faso and the UK. Findings will be reported through local community meetings, peer-reviewed publications and conference presentations.

Trial registration number

ClinicalTrials.gov identifier: NCT03705624

For peer review only - http://bmjopen.bmj.com/site/about/guidelines.xhtml

For peer review only

STRENGTHS AND LIMITATIONS OF THIS STUDY:

- This study evaluates for the first time the impact of enhanced community case management and monthly screening and treatment on the transmissibility of malaria from endemic populations to mosquitoes.
- The detailed examination of malaria infections, gametocyte production and infectivity to mosquitoes across two peak transmission seasons and the intervening low transmission season provides uniquely detailed information on the dynamics of parasite carriage and infectivity.
- Intensive monitoring of mosquito exposure in combination with repeated assessments of parasite carriage in the human population allows detailed assessments of the force of malaria infection at different time-points during the peak and low transmission season.
- A limitation of this current study is that findings from this setting where malaria transmission intensity is high, may not translate to areas of lower transmission intensity where the tested approaches may be used in malaria elimination efforts.

KEY WORDS:

Malaria, transmission, direct membrane feeding assays, gametocyte, asymptomatic infections

For peer review only

INTRODUCTION:

Malaria continues to be a major public health problem in sub Saharan Africa. In 2017, there were an estimated 435,000 deaths from malaria globally, 93% in sub Saharan Africa (1). Despite this sobering figure and indications that no measurable reduction in global malaria cases has been achieved since 2015 (1), many countries had experienced a considerable decline in malaria transmission prior to this (2, 3) meaning malaria elimination and eradication are back on the agenda of the international research community and policy makers (4, 5). To achieve this, interventions that specifically aim to reduce transmission of malaria parasites are high on the priority list of the research agenda for malaria eradication (6, 7). Transmission of malaria begins with the formation of sexual-stage malaria parasites — the gametocytes. In *P. falciparum* infection, gametocytes develop and mature in the bone marrow and first appear in the circulation 10-12 days after asexual parasites are detected (8). Upon release in circulation, male and female gametocytes may persist for several weeks after asexual parasites have been cleared. In chronic infections there is persistent (but fluctuating) production of gametocytes from their asexual progenitors (9). Once ingested by a blood feeding female *Anopheles* mosquito, male and female gametocytes activate into gametes that fertilize and ultimately render the mosquito infectious to humans. Gametocytes are present not only in symptomatic malaria cases but also in infections not accompanied by symptoms that are severe enough to elicit treatment-seeking behaviour — so-called 'asymptomatic infections' (10). Since these asymptomatic infections represent a large proportion of all infections present in malaria-endemic settings (11, 12), asymptomatic parasite carriage may be a major component of the human infectious reservoir for malaria (13-15). Therefore detecting and treating these infections could be a valuable approach for reducing transmission. It is unclear how many asymptomatic infections start off as symptomatic infections and could potentially be detected and treated by enhanced community case management. One scenario is that enhanced community case management could be used to prevent the majority of infectious days by abrogating infections before they become transmissible to mosquitoes. A more challenging scenario is that many incident infections never elicit symptoms and would therefore remain undetected even during enhanced community management with regular clinical examinations. In this situation, active screening approaches would be needed to identify asymptomatic infections for drug-based targeting to prevent or interrupt the production of infectious gametocytes. The number of infections acquired and the likelihood that infections give rise to clinical symptoms may be influenced by spatial and temporal variations in malaria exposure (16, 17). Thus, detailed entomological assessments may uncover small-scale variations in mosquito exposure that play a key role in determining the detectability and transmissibility of infections in a given area (18).

For peer review only - http://bmjopen.bmj.com/site/about/guidelines.xhtml

In this study, we evaluate the impact of two intensified diagnostic and treatment strategies on the human infectious reservoir of malaria, and compare these to the current standard of care. These diagnostic and treatment strategies aim to i) detect and treat symptomatic infections earlier and more comprehensively by enhanced community case management (CCM; weekly active screening for fever); and ii) detect and treat asymptomatic malaria infections by monthly screening with point-of-care rapid diagnostic tests (MSAT).

For peer review only

For peer review only - http://bmjopen.bmj.com/site/about/guidelines.xhtml

METHODS AND ANALYSIS:

Study design

This study is a cluster-randomised trial to determine the impact of two interventions on parasite carriage, gametocyte carriage and infectiousness to mosquitoes. A total of 180 compounds (consisting of 3-6 enrolled participants) will be randomised to one of three study arms (60 compounds per arm): arm 1 - current standard of care with passively monitored malaria infections; arm 2 - standard of care plus enhanced community case management (CCM); or arm 3 - standard of care and enhanced CCM, plus monthly screening and treatment (MSAT), as detailed in Table 1. The study will take place over approximately 18 months covering two high transmission seasons and the low transmission season (dry season) in between. Throughout the study, entomological monitoring will take place to evaluate the local mosquito population and potential exposure to infected mosquitoes.

Arm 1 (control): Standard of care
• Standard of care with passively monitored malaria incidence at local health facilities. Health facilities will be equipped with research staff and adequate supplies to ensure malaria diagnosis and treatment with the first line antimalarial artemether-lumefantrine (AL) according to national guidelines.<br>• Seasonal Malaria Chemoprevention in children <5 years of age implemented by the national malaria control programme (monthly treatment with sulfadoxine-pyrimethamine + amodiaquine from July to October).
Arm 2 (intervention): CCM
• Standard of care (as in arm 1)<br>• Seasonal Malaria Chemoprevention (as in arm 1)<br>• Enhanced Community Case Management for malaria (CCM) involving weekly active screening for fever using a research-grade thermometer by a trained health worker. A measured temperature ≥37.5°C or reported fever in the last 24 hours will prompt screening with a conventional rapid diagnostic test (RDT). RDT positive individuals will be treated with AL according to national guidelines
Arm 3 (intervention): CCM+MSAT
• Standard of care (as in arm 1 and 2)<br>• Seasonal Malaria Chemoprevention (as in arm 1 and 2)<br>• Enhanced CCM (as in arm 2)<br>• Monthly Screening and Treatment (MSAT) regardless of symptoms with a conventional RDT. Screening will be performed by research staff with 25-35 days between screening rounds; RDT positive individuals will be treated with AL according to national guidelines.

Table 1. Study arms

Gradual implementation of interventions

The aim of the study is to determine the ability of the interventions to detect malaria infections, and the transmission potential of these infections. To maximize coverage and ensure good adherence to intervention protocols the study will be implemented in two phases (Figure 1). Phase 1 will initiate prior to the first transmission season with 30 compounds enrolled per study arm. Phase 2 will initiate at the end of the first transmission season with an additional 30 compounds enrolled per study arm.

For peer review only - http://bmjopen.bmj.com/site/about/guidelines.xhtml

Parasitology data and mosquito infection data will be compiled and analysed before the start of the second transmission season (~May 2019), at which time sample size and study procedures may be amended upon ethical approval.

Study setting

This study will be conducted within the Saponé health and demographic surveillance system (HDSS) area, located 45 kilometres Southwest of Ouagadougou, Burkina Faso. This HDSS is part of the INDEPTH Network and covers a total population of 85,000 living in 10,841 compounds. Compounds are georeferenced and a census of the population is conducted annually. The population is served by 23 local health facilities and a district hospital. This is a region of intense and highly seasonal malaria transmission with peak transmission from approximately June to October. Asexual parasite prevalence by microscopy in this population is ~50% in children below 15 years of age (14) and studies in this and other malaria-endemic settings in Burkina Faso indicate that ~80% of all asexual parasite carriers have concurrent gametocytes at highly variable densities (14, 19).

Enrolment and randomization

The unit of randomisation in this study is a compound. Per compound a minimum of one and maximum of two members will be enrolled from each of the three age groups (under 5 years of age, 5 – 15 years of age, and over 15 years of age), so that each compound will provide 3-6 enrolled participants. All participants must be willing to use the designated local health facilities, and to accommodate indoor and outdoor mosquito sampling for the study duration. Inclusion and exclusion criteria for individual study participants are listed in Table 2. From the 2017 census data, an estimated 4,700 compounds in the Saponé HDSS contained the required number of participants in the each age category and are in principle eligible. Up to date census data will be used prior to the study to select eligible compounds for enrolment in the Sapone Marche and Pissy local health areas (Figure 2). 180 compounds will be enrolled and will be divided into 60 groups of 3 based on proximity. Within each group, one compound will be randomly allocated to each of the three study arms by a computer generated algorithm. To enable the impact of the interventions to be continually evaluated throughout the study period, the study is designed so that overall transmission intensity is not influenced. To ensure the force of infection remains high ≤50% of all compounds in a given area will be enrolled in the study. This will be achieved by combining each group of 3 enrolled compounds with 3 non-enrolled adjacent compounds to form a total of 60 groups (Figure 3). Simulations predict that the interventions will have very small impact on overall transmission in the study area relative to the current transmission intensity (Figure 4).

For peer review only - http://bmjopen.bmj.com/site/about/guidelines.xhtml

Inclusion criteria	
i.	Participants should be permanent residents of the compound
ii.	Participants should be willing to participate in repeated assessments of health and infection status and willing to donate a maximum of 37mL of blood (children <10 years of age) or 52mL of blood (older individuals) during an 18-month period
**Exclusion criteria**	
i.	Any (chronic) illness that would affect study participation
ii.	Pre-existing severe chronic health conditions
iii.	Current participation in malaria vaccine trials or participation in such trials in the last 2 years
iv.	History of intolerance to artemether-lumefantrine

Table 2. Inclusion and exclusion criteria for enrolled participants

Procedures

Study visits

At the start and end of each season, a cross-sectional survey will be performed concurrently in all enrolled compounds. These surveys will also include adults that are present in enrolled compounds but not participating in the interventions; additional informed consent will be obtained for this. In these surveys all individuals will have their axillary temperature measured and a finger prick blood sample taken for molecular assessment of gametocyte and parasite carriage (as detailed in Table 3.). In case of measured fever, the same finger prick blood sample will be used for malaria diagnosis by conventional rapid diagnostic test (RDT) and treatment will be offered as per national guidelines. Routine surveys will be conducted throughout the wet and dry seasons to assess parasite carriage, gametocyte carriage and infectivity to mosquitoes. These surveys will be performed on a subset of compounds each week on a rolling basis, so that each compound will visited every 10 weeks. Finger prick blood samples will be taken and processed as detailed in Table 3; one whole blood aliquot will be processed immediately for molecular detection of parasitemia by quantitative PCR (qPCR) targeting varATS (20). qPCR positive individuals (i.e. those with parasites, not necessarily gametocytes) will be invited to donate blood for mosquito feeding assays. The maximum number of feeding assays per day will be dependent on logistical feasibility. Asymptomatic infections that are detected by PCR will not be treated if unaccompanied by malaria symptoms, in line with national treatment guidelines. Routine surveys will continue throughout the dry season to allow a detailed assessment of the number of newly acquired infections when minimal exposure to infected mosquito bites is assumed.

In all three arms, clinical malaria episodes will be passively monitored at the local health facilities. Participants will be identified using study ID cards; for suspected malaria cases temperature and clinical history will be recorded. Fingerprick blood samples will be taken as detailed in Table 3. Malaria diagnosis will be performed by RDT and infected subjects will be treated with AL. For infections detected by community health workers during enhanced CCM (arm 2 and 3) or MSAT (arm

For peer review only - http://bmjopen.bmj.com/site/about/guidelines.xhtml

3) visits, the same procedure is used and samples are collected as detailed in Table 3. For RDT or microscopy positive infections detected at local health facilities or during the CCM or MSAT visits, when logistically feasible the individuals will be invited to donate a blood sample for mosquito feeding assays prior to treatment. For all mosquito feeding assays blood samples will be collected and transported in a portable incubator or thermos flask to the insectary for direct membrane feeding assays (DMFA).

Sample type/Procedure	Cross-sectional survey	Routine survey	Passive case detection	CCM	MSAT	DMFAs
Axillary temperature and clinical assessment	X	X	X	X	X	
Dried blood spot (DBS)	X	X	X	X*	X	
Whole blood in RNA protect	X	X	X	X*	X	X
Whole blood for DNA	X	X	X	X*	X	X
Rapid diagnostic test (RDT)	X*		X	X*	X	
Highly sensitive RDT (hsRDT)						X
Plasma						X
Blood smear			X#	X#	X#	X

Table 3. Sampling schedule: Table indicates the samples collected at each participant survey/visit. X* = sampling only takes place if subject has fever or history of fever in last 24 h; X# = sampling only takes place if subject has history of antimalarial treatment in previous 3 weeks for microscopic confirmation of malaria infection; CCM = enhanced community case management visit; MSAT = monthly screening and treatment visit; DMFA = direct membrane feeding assay.

Entomology

Detailed assessments of mosquito exposure will be performed at the compound level. Mosquito exposure will primarily be determined using a CDC miniature light trap (Model 512; John W. Hock Company, Gainesville, Florida, USA) placed inside one of the sleeping spaces close to participants sleeping under a net. Sampling will be performed in every compound once every 14 days, throughout the study. To explore outdoor biting and enable more sensitive sampling of sparse mosquito populations, Human Landing Catches (HLC) will be performed indoors and outdoors from dusk until dawn for a subset of compounds (21). Due to the low densities of mosquitoes present in the dry season, additional strategies will be used to optimise the potential for mosquito monitoring during that period. Dry season pyrethrum spray catches will be conducted in a selection of non-enrolled compounds to collect indoor resting mosquitoes. These compounds will be sprayed with the

For peer review only - http://bmjopen.bmj.com/site/about/guidelines.xhtml

For peer review only

insecticide aerosol Kaltox® (a mixture of pyrethroids and carbamate containing 0.27 % Allethrin, 0.2 % Tetramethrin, 0.17 % Permethrin, and 0.68 % Propoxur) for 30-45 seconds; dead and immobilized mosquitoes will be collected after 10 minutes. In addition, in the absence of water body larval habitats in the dry season an active search of refugia and resting trap collections may be performed close to study compounds. At all collections, factors such as housing type and presence of animals will be recorded and associations with mosquito exposure will be examined. Throughout the study, presence, size and location of water bodies will be periodically assessed.

Collected mosquitoes will be sorted and counted by sex and species using morphological characteristics, and classified according to blood feeding status and parity (unfed, blood-fed, semi-gravid and gravid) by microscopy. A subset of mosquitoes will be preserved for further molecular analysis (22, 23). Age structure of the mosquito population will be determined by examination of tracheal distension within the ovaries (24). Infection status of Anopheles caught in CDC traps will be determined using circumsporozoite ELISA (CSP-ELISA) (25, 26) and or PCR (27, 28).

Laboratory assessments

Direct membrane feeding assays (DMFA) will be performed to determine infectivity to locally reared female *Anopleles coluzzii* mosquitoes, as described previously (14). Whole blood collected in lithium heparin tubes will be offered in duplicate to 50-60 starved mosquitoes via an artificial membrane feeder (400-500 µl per feeder). Fully fed mosquitoes will be kept at 27-29°C and maintained on a sucrose diet for approximately one week (6 – 8 days). Mosquito infection will be determined by dissection and assessment for the presence and density of oocysts by two independent microscopists. Infected midguts will be stored for molecular confirmation of infection and genotyping for a subset of guts (14).

Parasite DNA and RNA will be extracted from whole blood samples collected in EDTA tubes. Parasitemia will be assessed using varATS qPCR (20) and gametocyte density and sex ratio will be assessed using established qRT-PCR protocols (29). Whole blood samples will be evaluated by HS-RDTs and presence and quantification of HRP2 will be also determined using the bead-based Luminex® (Austin, TX) platform (30). Multiplicity of infection will be determined in a selection of samples using MSP-genotyping (31) or equivalent techniques.

Red blood cell polymorphisms that are associated with the likelihood of developing blood-stage (clinical) malaria infection, the severity of malaria infection or gametocyte production (haemoglobin C, haemoglobin S, α-thalassaemia and glucose-6-dehydrogenase deficiency) (32) will be measured in baseline samples by multiplex luminex assay (33). In secondary analyses, antibody responses to parasite and gametocyte antigens will be determined by protein microarray (34) and multiplex bead-

For peer review only - http://bmjopen.bmj.com/site/about/guidelines.xhtml

based serological assays (35) and associated with parasite carriage, clinical symptoms and infectivity. For a selection of samples functional transmission reducing immunity will be assessed by standard membrane feeding assay (SMFA) using plasma samples collected at the time of field-based DMFA (36).

Objectives/endpoints

The main study objective is to determine the impact of the two diagnostic strategies on parasite carriage. The hypothesis is that the parasite prevalence and density are reduced in arms 2 and 3 compared to the standard of care. More infections will be detected early and may therefore be abrogated before they develop into chronic asymptomatic infections. As such the primary endpoint is parasite prevalence and density in each arm by molecular detection in the end of study cross-sectional survey. Secondary objectives and endpoints seek to evaluate the impact of the interventions after each season, and to define the contribution and infectivity of gametocytes in these infections, as detailed in Table 4. Exploratory objectives aim to understand the detectability and transmissibility of infections, by exploring i) relationships between parasitemia and transmission, and ii) associations between infectivity and host characteristics, parasite characteristics, or mosquito exposure (Table 4).

For peer review only

Primary objective/endpoint
1. Parasite prevalence and density between arms by molecular detection at the end of study cross-sectional survey. [Time Frame: Month 18 (end of second transmission season; January-February 2020)]
**Secondary objectives/endpoints**
1. Parasite prevalence and density by molecular detection at the end of year 1 cross-sectional survey. [Time Frame: Month 6 (end of first transmission season; January-February 2019)]<br>2. Parasite prevalence and density by molecular detection at the end of the dry season cross-sectional survey. [Time Frame: Month 12 (prior to second transmission season; June 2019)]<br>3. Gametocyte prevalence and or density by molecular methods at the end of study cross-sectional survey. [Time Frame: Month 18 (end of second transmission season; January-February 2020)]<br>4. Gametocyte prevalence and or density by molecular methods at the end of year 1 cross-sectional survey. [Time Frame: Month 6 (end of first transmission season; January-February 2019)]<br>5. Gametocyte prevalence and or density by molecular methods at the end of the dry season cross-sectional. [Time Frame: Month 12 (prior to second transmission season; June 2019)]<br>6. Gametocyte prevalence and or density by molecular methods among *P. falciparum* infections during all visits in the study. [Time Frame: Throughout study, an average of 18 months]<br>7. The number of incident infections/clinical incidence detected during CCM, MSAT and passive case detection. [Time Frame: Throughout study, an average of 18 months]<br>8. Infectivity to mosquitoes of *P. falciparum* infections [Time Frame: Throughout study, an average of 18 months]
**Exploratory objectives/endpoints:**
1. The detectability of infections by highly sensitive rapid diagnostic tests. [Time Frame: Throughout study, an average of 18 months]<br>2. The relationship between the proportion of infected mosquitoes and gametocyte density. [Time Frame: Throughout study, an average of 18 months]<br>3. The impact of infection characteristics on the transmissibility of infections to mosquitoes [Time Frame: Throughout study, an average of 18 months]<br>4. The impact of human host characteristics on the transmissibility of infections to mosquitoes [Time Frame: Throughout study, an average of 18 months]<br>5. Association between total parasite density and gametocyte density [Time Frame: Throughout study, an average of 18 months]<br>6. Malaria transmission potential based on measured gametocyte densities and the association between gametocyte density and mosquito infection rates [Time Frame: Throughout study, an average of 18 months]<br>7. Number of acquired clones based on genotyping [Time Frame: Throughout study, an average of 18 months]<br>8. The duration of infections in the dry season [Time Frame: Month 6-12, between end of season survey January/February 2019 and end of dry season survey June 2019]<br>9. To quantify mosquito exposure in relation to incident infections and transmission opportunities [Time Frame: Throughout study, an average of 18 months]

Table 4. Objectives and endpoints

CCM = enhanced community case management involving active weekly fever screening; MSAT = Monthly screening and treatment

For peer review only - http://bmjopen.bmj.com/site/about/guidelines.xhtml

Data analysis

For primary and secondary endpoints, parasite and gametocyte prevalence will be compared between arms using multivariate mixed effect logistic regression models; parasite and gametocyte density will be assessed using multivariate mixed effect linear regression models on log transformed values. The number of incident infections will be assessed by Poisson regression and compared between arms. Proportion of infectious participants and proportion of infected mosquitoes will be assessed by logistic regression models. Analyses will take account of correlation of outcomes at the compound level and the matched randomisation scheme. There may be issues with convergence in mixed effect models given the small number of subjects per compound. In this case analysis based on compound level summaries will be performed. For exploratory endpoints, detected infections will be analysed in relation to the time and duration of infection, parasite and gametocyte prevalence and density, symptoms, complexity of infection, host characteristics, mosquito exposure and infectiousness to mosquitoes. Parasite density will be related to detectability by (ultra-sensitive) rapid diagnostic tests. The association between gametocyte density and mosquito infection prevalence and intensity will be determined using an established Bayesian hierarchical model that incorporates the density of male gametocytes and gametocyte sex ratio (37). In exploratory endpoints, model fitting will be performed for different strata of infection characteristics (e.g. complexity of infection, concurrent asexual parasite density, and season) and host characteristics (e.g. anti-gametocyte immune responses, age). Mosquito exposure by indoor and outdoor trapping methods will be associated with the likelihoods of incident infections and opportunities to transmit infections using comprehensive malaria transmission models (38).

Sample size calculations and predicted impact of interventions

The hypothesis of the current study is that the parasite prevalence is considerably reduced in arms 2 and 3 compared to the standard of care. Assuming a conservative parasite prevalence of 40% in the control arm, the current sample size should be sufficient to detect a 2-fold reduction in parasite prevalence with >99% power. This sample size will also allow us to detect the anticipated difference in the proportion of infections that are gametocyte-positive at the moment of detection (secondary objectives 3-5). We assume that ≥80% of all infections detected in cross-sectional surveys (i.e. chronic asymptomatic infections) concurrently carry gametocytes (14). We expect that infections that are detected by CCM or MSAT visits in arms 2 and 3 are of shorter duration and have lower gametocyte prevalence and density (15). If we assume ≤50% gametocyte prevalence among these CCM/MSAT-detected infections, monitoring 100 survey-detected and 100 CCM/MSAT-detected infections will result in 99% power to detect this difference.

For peer review only - http://bmjopen.bmj.com/site/about/guidelines.xhtml

Simulations predict that enhanced CCM will reduce asexual parasite carriage in arm 2 by 70%, 60%, and 30% in children under 5, 5-15 year olds, and adults > 15 years, respectively. The addition of MSAT to arm 3 will further reduce asexual carriage in adults by 10% relative to arm 2 but have minimal impact in the other age groups (Figure 5). Simulations were carried out in EMOD v2.15 (39, 40) an agent-based model of malaria transmission using within-host infection, immunity, and infectivity dynamics (38, 41) calibrated to field data (42). The study site census was gridded into 1km grid cells with total human population of 7,800 individuals and birth and death rates of 39 per 1,000 per year, resulting in 17% of population being under 5 and 25% between 5 and 15. Within a grid cell, biting rate is surface area dependent but otherwise homogeneous. Humans moved between grid cells at a rate of one trip per person per year, and each trip lasted on average two days. Vectors did not move between grid cells. Malaria dynamics are simulated in a highly seasonal setting with annual EIR of 150 in the presence of ITNs. Seasonality was based on entomology data collected in 2011. The vector population was modeled as *An. gambiae* mosquitoes with 65% anthropophily and 90% indoor feeding. All grid cells had the same seasonality and transmission intensity. For the simulations, the number of individuals per arm were ~165 during phase 1 until January 2019 when numbers increased by ~200 individuals per arm for phase 2. Means of 500 stochastic realizations are shown. Simulated interventions are as follows:

1. **SMC –** Administration of a curative drug with prophylactic protection for 30 days to children < 5 years. Four rounds of SMC carried out each year starting June 30 with 30 days between rounds. Coverage was set at 80% per round and independent between rounds.

2. **ITNs – Estimated age dependent distribution;** 0-5y = 67%, 5-10y = 74%, 10-15y = 68%, 15-20y = 65%, and >20y = 71%.

3. **Passive case detection and health seeking behaviour –** AL administered to uncomplicated and severe malaria cases with age-dependent distance-based health seeking. Distances to the two study health facilities in Sapone and Pissy were calculated from the center of each grid cell with exponential half-distance in coverage of 10 km. Maximum coverage was 50% for children under 5, 30% for individuals over 5, and 80% for severe cases. Patients received treatment within 3 days of onset of clinical symptoms.

4. **Enhanced community case management (CCM) in arms 2 and 3 –** Modeled as continuous weekly active screening where fevers of 37.5°C or higher receive AL, beginning June 30, 2018 with 100% coverage.

For peer review only - http://bmjopen.bmj.com/site/about/guidelines.xhtml

5. **MSAT with RDT in arm 3 –** Modeled as continuous monthly RDT testing (diagnostic sensitivity 40 asexual parasites/µL), with RDT positive individuals receiving AL, beginning June 30, 2018 with 100% coverage.

ETHICS AND DISSEMINATION:

Ethical considerations

The purpose of the study and practical consequences of participation will be explained during both community meetings and visits to each household by experienced nurses. Objectives and procedures will be explained verbally and given in printed form in French or a local language. All compound members will be screened by a GCP-trained clinician for enrolment criteria, signs of acute disease, and individual informed consent. Malaria infections will not be cleared prior to inclusion in the study but this may be considered later based on results of the first season. Payment will not be given for participation but instead participants will receive financial compensation for travel and time. In addition, all participants will receive a free long-lasting insecticide treated bed net and treatment for malaria and other common illnesses free of charge. The blood draw volumes will be minimized: a maximum of 6 mL of blood will be taken at any single time-point and the total volume of blood taken during a 6 month period will be lower than 37 mL for individuals enrolled in arms 3, or lower than 22 mL for those in arms 1 and 2. Both the cumulative blood draw volume and maximum blood volume taken in a single sample are within the limits considered as physiological "minimal risk" (43). No experimental medication is used in this study; AL is approved in country as first-line antimalarial treatment. Study identification numbers will be used on all paper and electronic questionnaires. Some questionnaires will be programmed using OpenDataKit with data entered into password-secured, encrypted tablets. Data will be uploaded daily to an encrypted cloud-based sever. The study has been reviewed and approved by London School of Hygiene and Tropical Medicine (LSHTM) (Review number: 14724) and The Centre National de Recherche et de Formation sur le Paludisme institutional review board (IRB) (Deliberation N° 2018/000002/MS/SG/CNRFP/CIB) and Burkina Faso national medical ethics committees. (Deliberation N° 2018-01-010). The current protocol version is 2.0 approved November 2018. Any modifications to the protocol which may impact on the conduct of the study, subject safety or potential benefit of the subjects, including changes of study objectives, study design, study procedures, study population or sample size will require a formal amendment to the protocol. Such amendment will be approved by the Ethics Committees/IRB prior to implementation.

For peer review only - http://bmjopen.bmj.com/site/about/guidelines.xhtml

Trial oversight, monitoring and quality control

This study will comply with GCP guidelines and be conducted in accordance with the latest South Africa revision of the Declaration of Helsinki and local regulatory requirements. All study staff will receive a local workshop in Good Clinical Practice before the study starts. Since no experimental treatment is used in the study a Data Safety and Monitoring Committee will not be installed; a scientific steering committee will provide technical oversight and will assist with interpretation of data. LSHTM is the Research Sponsor, taking responsibility for the initiation and management of the trial clinical trial including trial monitoring. The LSHTM Research Governance and Integrity Office has reviewed, assessed and registered the trial and holds Public Liability ("negligent harm") and Clinical Trial ("non-negligent harm") insurance policies which apply to this trial.

Periodically, a subset of compounds will be randomly selected from each arm to be visited by the quality control team to monitor study implementation adherence and unintended effects of study interventions or conduct. Quality control for mosquito feeding assays includes regular enrolment of microscopy-positive gametocyte carriers as controls. For this, children aged 10-15 years from non-participating compounds will be screened for the presence of gametocytes by microscopy and up to 5 gametocyte carriers will be enrolled every 6 weeks. Specific informed consent will be provided for this.

Dissemination

Findings of the study will be shared with the community via local opinion leaders and community meetings. Results may also be shared through conferences, seminars, reports, theses, and peer-reviewed publications; disease occurrence data and study outcomes will be shared with the Ministry of Health. Data will be published in an online digital repository.

For peer review only

For peer review only - http://bmjopen.bmj.com/site/about/guidelines.xhtml

AKNOWLEDGMENTS:

The authors would like to thank the following individuals for their contributions to the study protocol development: Bronner Gonçalves, Umberto D'Alessandro, Edward Wenger, Lorenz von Seidlein & Tovi Lehman.

AUTHORS CONTRIBUTIONS:

AT, TB, CD and KC developed and designed the study. MG, AO, SA, IS, AN, PS, JG, JB and WS contributed to study design. All authors contributed to protocol development. KC, AO, CD, TB and AT wrote the manuscript. All authors reviewed and approved the manuscript final draft.

FUNDING STATEMENT:

This study is funded by Bill and Melinda Gates Foundation grant (OPP1173572). KC and TB are also supported by a fellowship from the European Research Council (ERC-2014-StG639776). AT is supported by an award from UK Medical Research Council (MRC) and the UK Department for International Development (DFID) under the MRC/DFID Concordat agreement (MR/P02016X/1)

COMPETING INTEREST STATEMENT:

All authors declare no competing interest.

ROLE OF FUNDING SOURCE

The funders, Bill &Melinda Gates Foundation, were involved in study design, but had no role in data collection, analysis, interpretation, or reporting.

For peer review only

REFERENCES:

1. World Health Organization (WHO). World Malaria Report 2018. https://www.who.int/malaria/publications/world-malaria-report-2018/en/: .
2. Bhattarai A, Ali AS, Kachur SP, et al. Impact of artemisinin-based combination therapy and insecticide-treated nets on malaria burden in Zanzibar. *PLoSMed*. 2007;4(11):e309.
3. Barnes KI, Durrheim DN, Little F, et al. Effect of artemether-lumefantrine policy and improved vector control on malaria burden in KwaZulu-Natal, South Africa. *PLoSMed*. 2005;2(11):e330.
4. Alonso PL, Brown G, Arevalo-Herrera M, et al. A research agenda to underpin malaria eradication. *PLoS Med*. 2011;8(1):e1000406.
5. Greenwood BM. Control to elimination: implications for malaria research. *Trends Parasitol*. 2008;24(10):449-54.
6. MalERA Consultative Group on Drugs. A research agenda for malaria eradication: drugs. *PLoS medicine*. 2011;8(1):e1000402.
7. MalERA Consultative Group on Vaccines. A research agenda for malaria eradication: vaccines. *PLoS medicine*. 2011;8(1):e1000398.
8. Eichner M, Diebner HH, Molineaux L, et al. Genesis, sequestration and survival of Plasmodium falciparum gametocytes: parameter estimates from fitting a model to malaria therapy data. *Trans R Soc Trop Med Hyg*. 2001;95.
9. Johnston GL, Smith DL, Fidock DA. Malaria's missing number: calculating the human component of R0 by a within-host mechanistic model of Plasmodium falciparum infection and transmission. *PLoS Comput Biol*. 2013;9(4):e1003025.
10. Bousema T, Drakeley C. Epidemiology and infectivity of Plasmodium falciparum and Plasmodium vivax gametocytes in relation to malaria control and elimination. *Clinical microbiology reviews*. 2011;24(2):377-410.
11. Imwong M, Stepniewska K, Tripura R, et al. Numerical Distributions of Parasite Densities During Asymptomatic Malaria. *J Infect Dis*. 2016;213(8):1322-9.
12. Bousema T, Okell L, Felger I, et al. Asymptomatic malaria infections: detectability, transmissibility and public health relevance. *Nature reviews Microbiology*. 2014;12(12):833-40.
13. Slater HC, Ross A, Ouedraogo AL, et al. Assessing the impact of next-generation rapid diagnostic tests on Plasmodium falciparum malaria elimination strategies. *Nature*. 2015;528(7580):S94-101.
14. Goncalves BP, Kapulu MC, Sawa P, et al. Examining the human infectious reservoir for Plasmodium falciparum malaria in areas of differing transmission intensity. *Nat Commun*. 2017;8(1):1133.
15. Tadesse FG, Slater HC, Chali W, et al. The relative contribution of symptomatic and asymptomatic Plasmodium vivax and Plasmodium falciparum infections to the infectious reservoir in a low-endemic setting in Ethiopia. *Clin Infect Dis*. 2018.
16. Bousema T, Griffin JT, Sauerwein RW, et al. Hitting hotspots: spatial targeting of malaria for control and elimination. *PLoS medicine*. 2012;9(1):e1001165.
17. Bejon P, Williams TN, Liljander A, et al. Stable and unstable malaria hotspots in longitudinal cohort studies in Kenya. *PLoS medicine*. 2010;7(7):e1000304.
18. Rodriguez-Barraquer I, Arinaitwe E, Jagannathan P, et al. Quantification of anti-parasite and anti-disease immunity to malaria as a function of age and exposure. *eLife*. 2018;7.
19. Ouedraogo AL, Schneider P, de Kruijf M, et al. Age-dependent distribution of Plasmodium falciparum gametocytes quantified by Pfs25 real-time QT-NASBA in a cross-sectional study in Burkina Faso. *AmJTropMedHyg*. 2007;76(4):626-30.
20. Hofmann N, Mwingira F, Shekalaghe S, et al. Ultra-sensitive detection of Plasmodium falciparum by amplification of multi-copy subtelomeric targets. *PLoS medicine*. 2015;12(3):e1001788.
21. Riehle MM, Guelbeogo WM, Gneme A, et al. A cryptic subgroup of Anopheles gambiae is highly susceptible to human malaria parasites. *Science*. 2011;331(6017):596-8.

For peer review only - http://bmjopen.bmj.com/site/about/guidelines.xhtml

22. Koekemoer LL, Kamau L, Hunt RH, et al. A cocktail polymerase chain reaction assay to identify members of the Anopheles funestus (Diptera: Culicidae) group. *Am J Trop Med Hyg*. 2002;66(6):804-11.
23. Santolamazza F, Calzetta M, Etang J, et al. Distribution of knock-down resistance mutations in Anopheles gambiae molecular forms in west and west-central Africa. *Malar J*. 2008;7:74.
24. Detinova TS. Determination of the physiological age of Anopheles females by the changes in the tracheal system of the ovaries. *Med Parazitol (Mosk)*. 1945;14(2):45-9.
25. Wirtz RA, Burkot TR, Graves PM, et al. Field evaluation of enzyme-linked immunosorbent assays for Plasmodium falciparum and Plasmodium vivax sporozoites in mosquitoes (Diptera: Culicidae) from Papua New Guinea. *Journal of medical entomology*. 1987;24(4):433-7.
26. Wirtz RA, Duncan JF, Njelesani EK, et al. ELISA method for detecting Plasmodium falciparum circumsporozoite antibody. *Bulletin of the World Health Organization*. 1989;67(5):535-42.
27. Snounou G, Viriyakosol S, Zhu XP, et al. High sensitivity of detection of human malaria parasites by the use of nested polymerase chain reaction. *Molecular and biochemical parasitology*. 1993;61(2):315-20.
28. Graumans W, Tadesse FG, Andolina C, et al. Semi-high-throughput detection of Plasmodium falciparum and Plasmodium vivax oocysts in mosquitoes using bead-beating followed by circumsporozoite ELISA and quantitative PCR. *Malar J*. 2017;16(1):356.
29. Meerstein-Kessel L, Andolina C, Carrio E, et al. A multiplex assay for the sensitive detection and quantification of male and female Plasmodium falciparum gametocytes. *Malar J*. 2018;17(1):441.
30. Plucinski MM, Dimbu PR, Fortes F, et al. Post-Treatment HRP2 Clearance in Patients with Uncomplicated Plasmodium falciparum Malaria. *J Infect Dis*. 2017.
31. Felger I, Maire M, Bretscher MT, et al. The dynamics of natural Plasmodium falciparum infections. *PLoS One*. 2012;7(9):e45542.
32. Lopera-Mesa TM, Doumbia S, Konate D, et al. Effect of red blood cell variants on childhood malaria in Mali: a prospective cohort study. *Lancet Haematol*. 2015;2(4):e140-9.
33. Grignard L, Mair C, Curry J, et al. Bead-based assays to simultaneously detect multiple human inherited blood disorders associated with malaria. *Malar J*. 2019;18(1):14.
34. Stone WJR, Campo JJ, Ouedraogo AL, et al. Unravelling the immune signature of Plasmodium falciparum transmission-reducing immunity. *Nat Commun*. 2018;9(1):558.
35. Varela ML, Mbengue B, Basse A, et al. Optimization of a magnetic bead-based assay (MAGPIX((R))-Luminex) for immune surveillance of exposure to malaria using multiple Plasmodium antigens and sera from different endemic settings. *Malar J*. 2018;17(1):324.
36. Ponnudurai T, Lensen AH, Van Gemert GJ, et al. Infectivity of cultured Plasmodium falciparum gametocytes to mosquitoes. *Parasitology*. 1989;98 Pt 2:165-73.
37. Bradley J, Stone W, Da D, et al. Predicting the likelihood and intensity of mosquito infection from sex specific Plasmodium falciparum gametocyte density *eLife*. 2018;7: pii: e34463.
38. Selvaraj P, Wenger EA, Gerardin J. Seasonality and heterogeneity of malaria transmission determine success of interventions in high-endemic settings: a modeling study. *BMC infectious diseases*. 2018;18(1):413.
39. Eckhoff PA. A malaria transmission-directed model of mosquito life cycle and ecology. *Malar J*. 2011;10:303.
40. Bershteyn A, Gerardin J, Bridenbecker D, et al. Implementation and applications of EMOD, an individual-based multi-disease modeling platform. *Pathog Dis*. 2018.
41. Eckhoff P. P. falciparum infection durations and infectiousness are shaped by antigenic variation and innate and adaptive host immunity in a mathematical model. *PLoS One*. 2012;7(9):e44950.
42. Ouedraogo AL, Goncalves BP, Gneme A, et al. Dynamics of the Human Infectious Reservoir for Malaria Determined by Mosquito Feeding Assays and Ultrasensitive Malaria Diagnosis in Burkina Faso. *J Infect Dis*. 2016;213(1):90-9.
43. Howie SR. Blood sample volumes in child health research: review of safe limits. *Bulletin of the World Health Organization*. 2011;89(1):46-53.

For peer review only - http://bmjopen.bmj.com/site/about/guidelines.xhtml

FIGURES LEGENDS:

Figure 1. Study flow diagram

Compounds are enrolled into the study during 2 Phases of recruitment and randomised to the three study arms. Enhanced CCM = Community case management, MSAT = Monthly screening and treatment, LHF = local health facility.

Figure 2. Map of Sapone HDSS

Sapone Health and Demographic Surveillance System (HDSS) area with selected study area in highlighted in orange

Figure 3. Study enrolment strategy

Health and demographic surveillance system census data was used to select eligible compounds based on the number and age of occupants. Eligible compounds were enrolled and groups of 3 enrolled compounds were combined with 3 non-enrolled compounds, generating 60 groups. Within each group the 3 enrolled compounds were randomly assigned to each of the 3 study arms. This strategy is used to ensure 1) an even spread or compounds in each arm over the study area and, 2) that ≤50% of all compounds in the study area are enrolled in the study, so overall transmission intensity remains unaffected.

Figure 4. Interventions have minimal impact on overall transmission in the study area relative to transmission intensity

True prevalence of any malaria infection in simulations with INDIE interventions (red) and without (blue) is similar. Simulated INDIE activities begin in July 2018 and continue through the end of simulation. Lines indicate mean and shaded area the 95% observed interval across 25 stochastic realizations.

Figure 5. Simulations predict impact of interventions in arms 2 and 3 across age groups

Simulation of the impact of enhanced CCM (arm 2, green) and enhanced CCM+MSAT (arm 3, orange) relative to no intervention (arm 1, purple) on parasite carriage in young children, school-age children, and adults. Lines indicate mean and shaded area the 95% observed interval of prevalence by PCR with sensitivity 4 parasites/µL across 500 stochastic realizations.

For peer review only - http://bmjopen.bmj.com/site/about/guidelines.xhtml

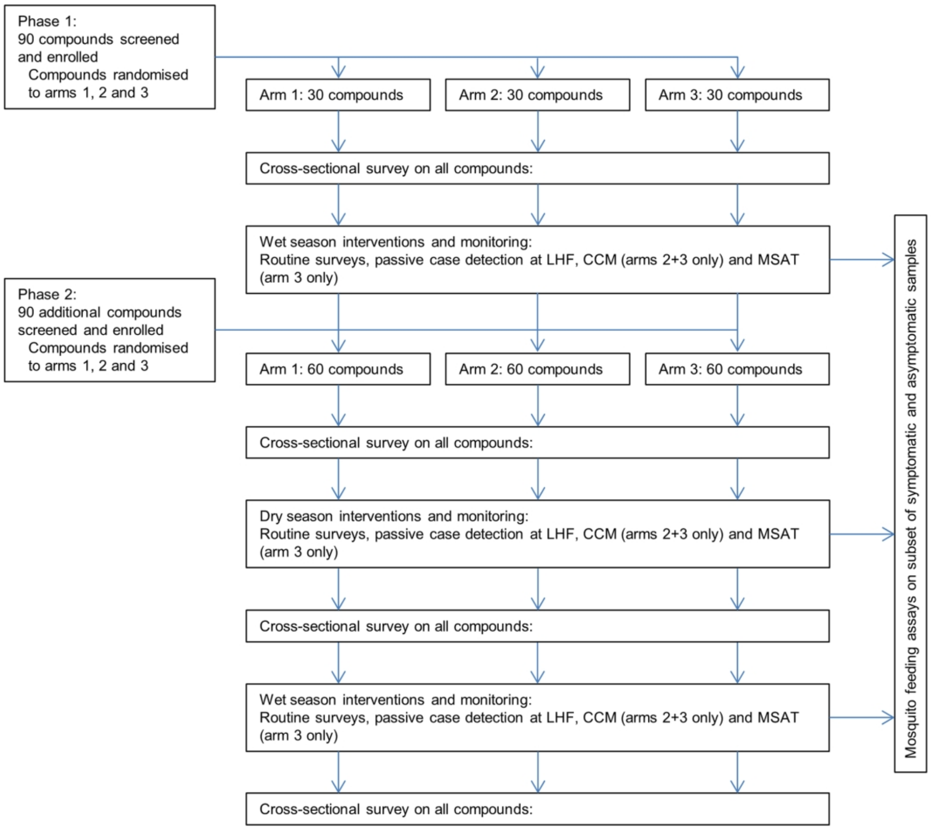

Figure 1

190x162mm (300 x 300 DPI)

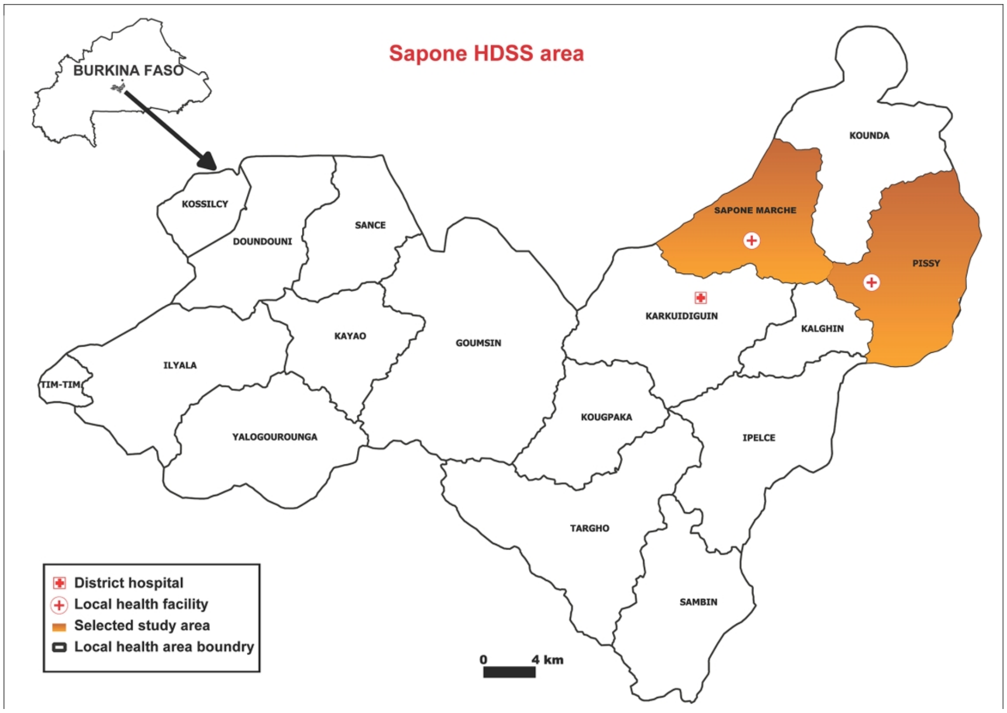
Sapone HDSS area
BURKINA FASO
KOSSILCY
DOUNDOUNI
SANCE
KAYAO
GOUMSIN
ILYALA
TIM-TIM
YALOGOUROUNGA
KOUGPAKA
TARGHO
SAMBIN
IPELCE
KARKUIDIGUIN
KALGHIN
SAPONE MARCHE
KOUNDA
PISSY
District hospital
Local health facility
Selected study area
Local health area boundry
0
4 km

Figure 2

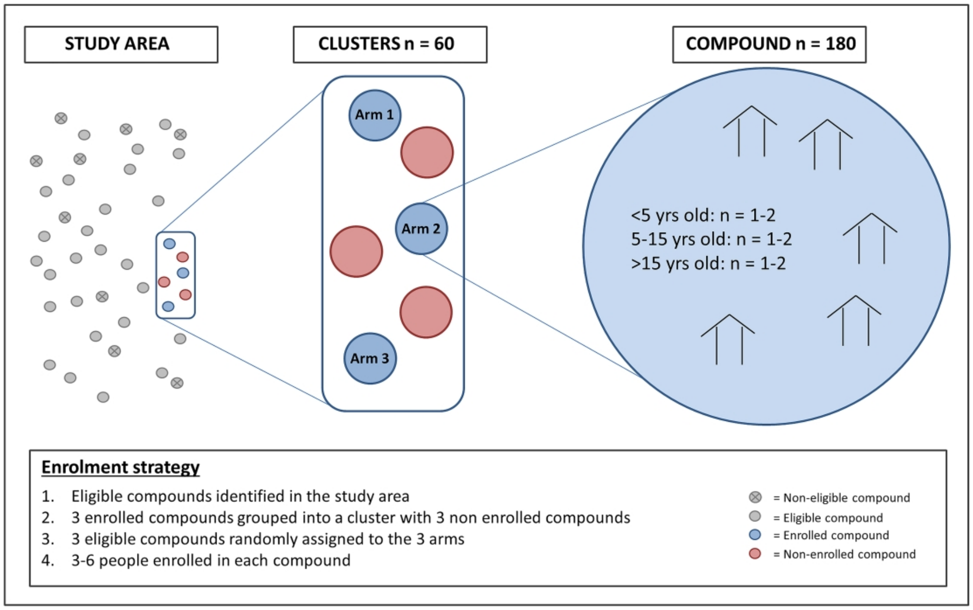
STUDY AREA
CLUSTERS n = 60
COMPOUND n = 180
Arm 1
Arm 2
Arm 3
<5 yrs old: n = 1-2
5-15 yrs old: n = 1-2
>15 yrs old: n = 1-2
Enrolment strategy
1. Eligible compounds identified in the study area
2. 3 enrolled compounds grouped into a cluster with 3 non enrolled compounds
3. 3 eligible compounds randomly assigned to the 3 arms
4. 3-6 people enrolled in each compound
= Non-eligible compound
= Eligible compound
= Enrolled compound
= Non-enrolled compound

Figure 3

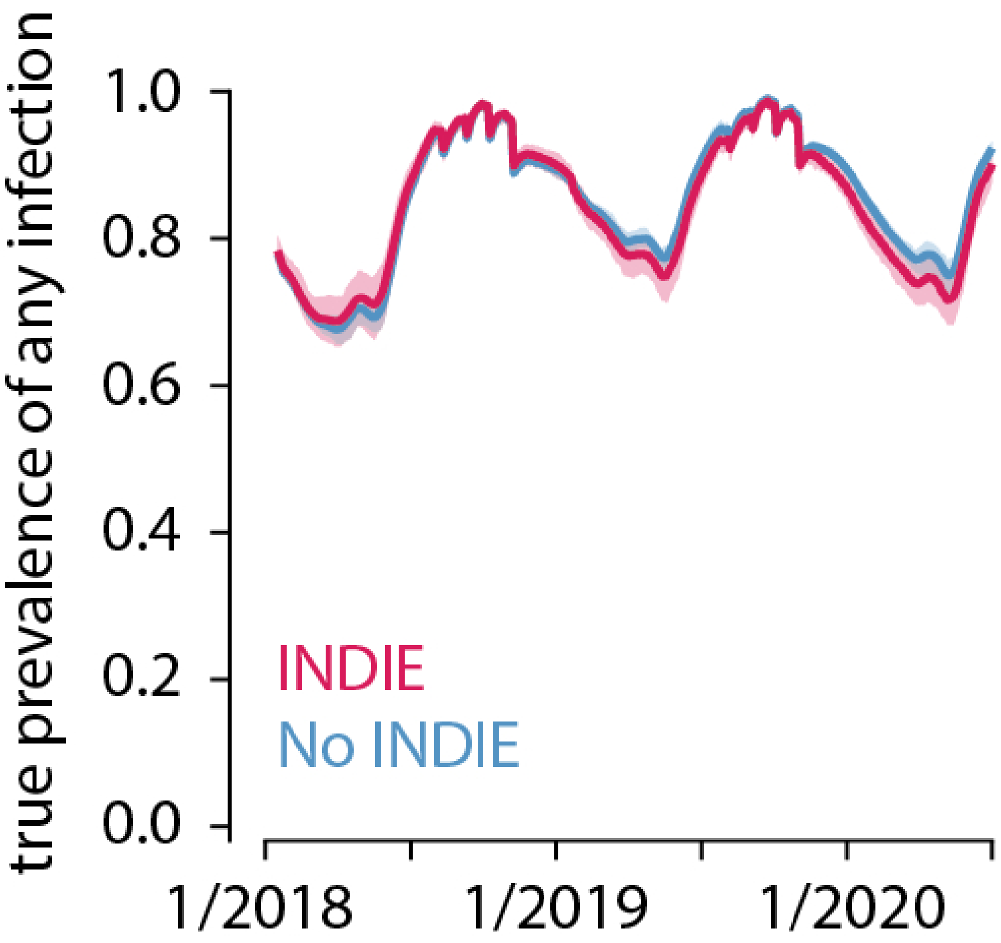
true prevalence of any infection
1.0
0.8
0.6
0.4
0.2
0.0
INDIE
No INDIE
1/2018
1/2019
1/2020

Figure 4

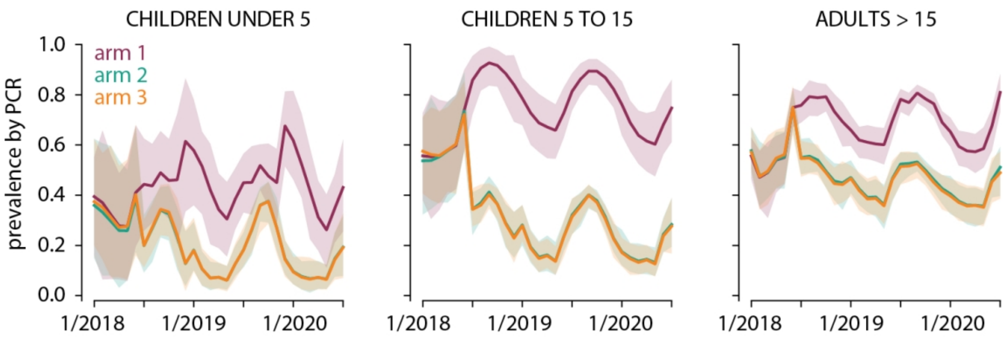
CHILDREN UNDER 5
CHILDREN 5 TO 15
ADULTS > 15
arm 1
arm 2
arm 3
prevalence by PCR
1.0
0.8
0.6
0.4
0.2
0.0
1/2018
1/2019
1/2020

Figure 5

Supplementary Information

Data category	Information
**Primary registry and trial identifying number**	ClinicalTrials.gov: NCT03705624
**Date of registration in primary registry**	July 19, 2018
**Secondary identifying numbers**	INDIE-1a
**Source(s) of monetary or material support**	The Bill and Melinda Gates Foundation
**Primary sponsor**	London School of Hygiene and Tropical Medicine<br>Keppel Street<br>London WC1E 7HT<br>Tel: +44 207 927 2626<br>Email: RGIO@lshtm.ac.uk
**Secondary sponsor(s)**	N/A
**Contact for public queries**	TeunBousema, PhD +31 0243617574 (teun.bousema@radboudumc.nl)<br>Katharine Collins, PhD +31 0243613663 (katharine.collins@radboudumc.nl)<br>Chris Drakeley, PhD +44 02079272289 (chris.drakeley@lshtm.ac.uk)<br>Alfred Tiono, MD, PhD +226 50324695 (t.alfred@fasonet.bf)
**Contact for scientific queries**	TeunBousema, PhD +31 243617574 (teun.bousema@radboudumc.nl)<br>Chris Drakeley, PhD +44 2079272289 (chris.drakeley@lshtm.ac.uk)<br>Alfred Tiono, MD, PhD +226 50324695 (t.alfred@fasonet.bf)<br>Katharine Collins, PhD+31 243613663 (katharine.collins@radboudumc.nl)
**Public title**	P. falciparum Infection Dynamics and Transmission to Inform Elimination (INDIE 1a)
**Scientific title**	A cluster-randomized trial investigating the impact of enhanced community case management and monthly screening and treatment on the transmissibility of malaria infections in Burkina Faso
**Countries of recruitment**	Burkina Faso
**Health condition(s) or problem(s) studied**	Malaria
**Intervention(s)**	<u>Arm 1 (control): Standard of care</u><br>• Standard of care with passively monitored malaria incidence<br><u>Arm 2 (intervention): CCM</u><br>• Standard of care plus enhanced Community Case Management for Malaria (CCM) involving weekly active screening for fever. A measured temperature ≥37.5°C or reported fever in last 24h will prompt screening with a conventional rapid diagnostic test (RDT). RDT positive individuals will be treated with artemether-lumefantrine according to national guidelines<br><u>Arm 3 (intervention): CCM + MSAT</u><br>• Standard of care with enhanced CCM plus monthly screening and treatment regardless of symptoms using a conventional RDT (MSAT). RDT positive individuals will be treated with artemether-lumefantrine according to national guidelines.
**Key inclusion and exclusion criteria**	<u>Inclusion Criteria:</u><br>1. Participants should be permanent residents of the compound<br>2. Participants should be willing to participate in repeated assessments of health and infection status and willing to donate a maximum of 37mL of blood (children <10 years of age) or 52mL of blood (older individuals) during an 18-month period

For peer review only - http://bmjopen.bmj.com/site/about/guidelines.xhtml

	Exclusion Criteria:<br>1. Any (chronic) illness that would affect with study participation<br>2. Pre-existing severe chronic health conditions<br>3. Current participation in malaria vaccine trials or participation in such trials in the last 2 years<br>4. History of intolerance to artemether-lumefantrine
**Study type**	Interventional<br>Allocation: randomized<br>Intervention model: Cluster randomized trial<br>Masking: none (open label)<br>Primary purpose: Diagnostic
**Date of first enrolment**	June 2018
**Target sample size**	900
**Recruitment status**	Recruiting
**Primary outcome(s)**	Parasite prevalence and density between arms by molecular detection at the end of study cross-sectional survey [Time Frame: Month 18 (end of second transmission season; January-February 2020)].
**Key secondary outcomes**	1. Parasite prevalence and density by molecular detection at the end of year 1 cross-sectional survey [Time Frame: Month 6 (end of first transmission season; January-February 2019)].<br>2. Parasite prevalence and density by molecular detection at the end of the dry season cross-sectional survey [Time Frame: Month 12 (prior to second transmission season; June 2019)].<br>3. Gametocyte prevalence and or density by molecular methods at the end of study cross-sectional survey [Time Frame: Month 18 (end of second transmission season; January-February 2020)].<br>4. Gametocyte prevalence and or density by molecular methods at the end of year 1 cross-sectional survey [Time Frame: Month 6 (end of first transmission season; January-February 2019)].<br>5. Gametocyte prevalence and or density by molecular methods at the end of the dry season cross-sectional [Time Frame: Month 12 (prior to second transmission season; June 2019)].<br>6. Gametocyte prevalence and or density by molecular methods among *P. falciparum* infections during all visits in the study [Time Frame: Throughout study, an average of 18 months].<br>7. The number of incident infections/clinical incidence detected during CCM, MSAT and passive case detection [Time Frame: Throughout study, an average of 18 months].<br>8. Infectivity to mosquitoes of *P. falciparum* infections [Time Frame: Throughout study, an average of 18 months].

Table S1. World Health Organization Trial Registration Data Set

For peer review only

STANDARD PROTOCOL ITEMS: RECOMMENDATIONS FOR INTERVENTIONAL TRIALS

SPIRIT 2013 Checklist: Recommended items to address in a clinical trial protocol and related documents*

Section/item	Item No	Description	Addressed on page number
**Administrative information**			
Title	1	Descriptive title identifying the study design, population, interventions, and, if applicable, trial acronym	___1__________
Trial registration	2a	Trial identifier and registry name. If not yet registered, name of intended registry	___2__________
	2b	All items from the World Health Organization Trial Registration Data Set	___S1+2______
Protocol version	3	Date and version identifier	___15_________
Funding	4	Sources and types of financial, material, and other support	___17________
Roles and responsibilities	5a	Names, affiliations, and roles of protocol contributors	___17________
	5b	Name and contact information for the trial sponsor	___S1________
	5c	Role of study sponsor and funders, if any, in study design; collection, management, analysis, and interpretation of data; writing of the report; and the decision to submit the report for publication, including whether they will have ultimate authority over any of these activities	___17________
	5d	Composition, roles, and responsibilities of the coordinating centre, steering committee, endpoint adjudication committee, data management team, and other individuals or groups overseeing the trial, if applicable (see Item 21a for data monitoring committee)	___16_____

For peer review only

Introduction

Background and rationale	6a	Description of research question and justification for undertaking the trial, including summary of relevant studies (published and unpublished) examining benefits and harms for each intervention	__4+5_______
	6b	Explanation for choice of comparators	__4+5________
Objectives	7	Specific objectives or hypotheses	__11+12______
Trial design	8	Description of trial design including type of trial (eg, parallel group, crossover, factorial, single group), allocation ratio, and framework (eg, superiority, equivalence, noninferiority, exploratory)	__6+7________

Methods: Participants, interventions, and outcomes

Study setting	9	Description of study settings (eg, community clinic, academic hospital) and list of countries where data will be collected. Reference to where list of study sites can be obtained	__7_________
Eligibility criteria	10	Inclusion and exclusion criteria for participants. If applicable, eligibility criteria for study centres and individuals who will perform the interventions (eg, surgeons, psychotherapists)	__7+8_________
Interventions	11a	Interventions for each group with sufficient detail to allow replication, including how and when they will be administered	__6, 8+9______
	11b	Criteria for discontinuing or modifying allocated interventions for a given trial participant (eg, drug dose change in response to harms, participant request, or improving/worsening disease)	__N/A________
	11c	Strategies to improve adherence to intervention protocols, and any procedures for monitoring adherence (eg, drug tablet return, laboratory tests)	__6, 15+16____
	11d	Relevant concomitant care and interventions that are permitted or prohibited during the trial	__6________
Outcomes	12	Primary, secondary, and other outcomes, including the specific measurement variable (eg, systolic blood pressure), analysis metric (eg, change from baseline, final value, time to event), method of aggregation (eg, median, proportion), and time point for each outcome. Explanation of the clinical relevance of chosen efficacy and harm outcomes is strongly recommended	__11+12________
Participant timeline	13	Time schedule of enrolment, interventions (including any run-ins and washouts), assessments, and visits for participants. A schematic diagram is highly recommended (see Figure)	___Figure 1___

For peer review only - http://bmjopen.bmj.com/site/about/guidelines.xhtml

Sample size	14	Estimated number of participants needed to achieve study objectives and how it was determined, including clinical and statistical assumptions supporting any sample size calculations	__13________
Recruitment	15	Strategies for achieving adequate participant enrolment to reach target sample size	__6__________

Methods: Assignment of interventions (for controlled trials)

Allocation:

Sequence generation	16a	Method of generating the allocation sequence (eg, computer-generated random numbers), and list of any factors for stratification. To reduce predictability of a random sequence, details of any planned restriction (eg, blocking) should be provided in a separate document that is unavailable to those who enrol participants or assign interventions	__7_________
Allocation concealment mechanism	16b	Mechanism of implementing the allocation sequence (eg, central telephone; sequentially numbered, opaque, sealed envelopes), describing any steps to conceal the sequence until interventions are assigned	__N/A_________
Implementation	16c	Who will generate the allocation sequence, who will enrol participants, and who will assign participants to interventions	__N/A_________
Blinding (masking)	17a	Who will be blinded after assignment to interventions (eg, trial participants, care providers, outcome assessors, data analysts), and how	__N/A_________
	17b	If blinded, circumstances under which unblinding is permissible, and procedure for revealing a participant's allocated intervention during the trial	__N/A_________

Methods: Data collection, management, and analysis

Data collection methods	18a	Plans for assessment and collection of outcome, baseline, and other trial data, including any related processes to promote data quality (eg, duplicate measurements, training of assessors) and a description of study instruments (eg, questionnaires, laboratory tests) along with their reliability and validity, if known. Reference to where data collection forms can be found, if not in the protocol	___8+9_______
	18b	Plans to promote participant retention and complete follow-up, including list of any outcome data to be collected for participants who discontinue or deviate from intervention protocols	___15_______

Data management	19	Plans for data entry, coding, security, and storage, including any related processes to promote data quality (eg, double data entry; range checks for data values). Reference to where details of data management procedures can be found, if not in the protocol	___15+16_______
Statistical methods	20a	Statistical methods for analysing primary and secondary outcomes. Reference to where other details of the statistical analysis plan can be found, if not in the protocol	___13_____
	20b	Methods for any additional analyses (eg, subgroup and adjusted analyses)	___13________
	20c	Definition of analysis population relating to protocol non-adherence (eg, as randomised analysis), and any statistical methods to handle missing data (eg, multiple imputation)	___N/A________
**Methods: Monitoring**			
Data monitoring	21a	Composition of data monitoring committee (DMC); summary of its role and reporting structure; statement of whether it is independent from the sponsor and competing interests; and reference to where further details about its charter can be found, if not in the protocol. Alternatively, an explanation of why a DMC is not needed	___16______
	21b	Description of any interim analyses and stopping guidelines, including who will have access to these interim results and make the final decision to terminate the trial	___N/A________
Harms	22	Plans for collecting, assessing, reporting, and managing solicited and spontaneously reported adverse events and other unintended effects of trial interventions or trial conduct	___15+16_______
Auditing	23	Frequency and procedures for auditing trial conduct, if any, and whether the process will be independent from investigators and the sponsor	___15+16_______
**Ethics and dissemination**			
Research ethics approval	24	Plans for seeking research ethics committee/institutional review board (REC/IRB) approval	___15_______
Protocol amendments	25	Plans for communicating important protocol modifications (eg, changes to eligibility criteria, outcomes, analyses) to relevant parties (eg, investigators, REC/IRBs, trial participants, trial registries, journals, regulators)	___15________

Consent or assent	26a	Who will obtain informed consent or assent from potential trial participants or authorised surrogates, and how (see Item 32)	__15_______
	26b	Additional consent provisions for collection and use of participant data and biological specimens in ancillary studies, if applicable	__N/A________
Confidentiality	27	How personal information about potential and enrolled participants will be collected, shared, and maintained in order to protect confidentiality before, during, and after the trial	__15________
Declaration of interests	28	Financial and other competing interests for principal investigators for the overall trial and each study site	__17________
Access to data	29	Statement of who will have access to the final trial dataset, and disclosure of contractual agreements that limit such access for investigators	__N/A________
Ancillary and post-trial care	30	Provisions, if any, for ancillary and post-trial care, and for compensation to those who suffer harm from trial participation	__15_______
Dissemination policy	31a	Plans for investigators and sponsor to communicate trial results to participants, healthcare professionals, the public, and other relevant groups (eg, via publication, reporting in results databases, or other data sharing arrangements), including any publication restrictions	__16_________
	31b	Authorship eligibility guidelines and any intended use of professional writers	__N/A________
	31c	Plans, if any, for granting public access to the full protocol, participant-level dataset, and statistical code	__16________
**Appendices**			
Informed consent materials	32	Model consent form and other related documentation given to participants and authorised surrogates	__N/A________
Biological specimens	33	Plans for collection, laboratory evaluation, and storage of biological specimens for genetic or molecular analysis in the current trial and for future use in ancillary studies, if applicable	__N/A________

*It is strongly recommended that this checklist be read in conjunction with the SPIRIT 2013 Explanation & Elaboration for important clarification on the items. Amendments to the protocol should be tracked and dated. The SPIRIT checklist is copyrighted by the SPIRIT Group under the Creative Commons "Attribution-NonCommercial-NoDerivs 3.0 Unported" license.

BMJ Open

Study protocol for a cluster-randomized trial investigating the impact of enhanced community case management and monthly screening and treatment on the transmissibility of malaria infections in Burkina Faso

Journal:	*BMJ Open*
Manuscript ID	bmjopen-2019-030598.R1
Article Type:	Protocol
Date Submitted by the Author:	05-Aug-2019
Complete List of Authors:	Collins, Katharine; Radboudumc, Department or Medical Microbiology<br>Ouedraogo, Alphonse; Centre National de Recherche et de Formation sur le Paludisme<br>Guelbeogo, Wamdaogo ; Centre National de Recherche et de Formation sur le Paludisme<br>Awandu, Shehu; Radboudumc, Department or Medical Microbiology<br>Stone, Will; London School of Hygiene and Tropical Medicine, Department of Immunology and Infection<br>Soulama, Issiaka; Centre National de Recherche et de Formation sur le Paludisme<br>Ouattara, Maurice ; Centre National de Recherche et de Formation sur le Paludisme<br>Nombre, Apollinaire ; Centre National de Recherche et de Formation sur le Paludisme<br>Diarra, Amidou; Centre National de Recherche et de Formation sur le Paludisme<br>Bradley, John; London School of Hygiene and Tropical Medicine, MRC Tropical Epidemiology Group<br>Selvaraj, Prashanth; Institute for Disease Modeling<br>Gerardin, Jaline; Institute for Disease Modeling<br>Drakeley, Chris; London School of Hygiene and Tropical Medicine, Department of Immunology and Infection<br>Bousema, Teun; Radboudumc, Department or Medical Microbiology<br>Tiono, Alfred; Centre National de Recherche et de Formation sur le Paludisme
**Primary Subject Heading**:	Infectious diseases
Secondary Subject Heading:	Epidemiology, Global health, Infectious diseases
Keywords:	Malaria, Transmission, Gametocyte, Direct membrane feeding assay, asymptomatic infection, Plasmodium falciparum

TITLE:

Study protocol for a cluster-randomized trial investigating the impact of enhanced community case management and monthly screening and treatment on the transmissibility of malaria infections in Burkina Faso

AUTHORS:

Katharine A Collins[1*], Alphonse Ouedraogo[2*], Wamdaogo Moussa Guelbeogo[2], Shehu S. Awandu[1], Will Stone[3], Issiaka Soulama[2], Maurice S. Ouattara[2], Apollinaire Nombre[2], Amidou Diarra[2], John Bradley[4], Prashanth Selvaraj[5], Jaline Gerardin[5], Chris Drakeley[3^#], Teun Bousema[1^#], Alfred B. Tiono[2#]

AFFILIATIONS:

1. Department of Medical Microbiology, Radboud university medical center, Nijmegen, The Netherlands

2. Centre National de Recherche et de Formation sur le Paludisme, Ouagadougou , Burkina Faso

3. Department of Immunology and Infection, London School of Hygiene and Tropical Medicine, Faculty of Infectious and Tropical Diseases, London, United Kingdom

4. MRC Tropical Epidemiology Group, London School of Hygiene and Tropical Medicine, London, United Kingdom

5. Institute for Disease Modeling, Bellevue WA United States

[*] These authors contributed equally

[#] These authors contributed equally

[^]Corresponding authors:
Chris Drakeley. Department of Infection & Immunity, London School of Hygiene & Tropical Medicine, London, UK, Tel: +44 (0) 20 7927 2289, Email: chris.drakeley@lshtm.ac.uk

Teun Bousema. Department of Medical Microbiology, Radboud university medical center, Nijmegen, The Netherlands, Tel: +31 243617574, Email: teun.bousema@radboudumc.nl

For peer review only

For peer review only - http://bmjopen.bmj.com/site/about/guidelines.xhtml

ABSTRACT:

Introduction

A large proportion of malaria-infected individuals in endemic areas do not experience symptoms that prompt treatment-seeking. These asymptomatically infected individuals may retain their infections for many months during which sexual-stage parasites (gametocytes) are produced that may be transmissible to mosquitoes. Reductions in malaria transmission could be achieved by detecting and treating these infections early. This study assesses the impact of enhanced community case management and monthly screening and treatment on the prevalence and transmissibility of malaria infections.

Methods and analysis

This cluster-randomized trial will take place in Sapone, an area of intense, highly seasonal malaria in Burkina Faso. In total, 180 compounds will be randomized to 1 of 3 interventions: arm 1 - current standard of care with passively monitored malaria infections; arm 2 - standard of care plus enhanced community case management (CCM), comprising active weekly screening for fever, and detection and treatment of infections in fever positive individuals using conventional rapid diagnostic tests (RDTs); or arm 3 - standard of care and enhanced CCM, plus monthly screening and treatment (MSAT) using RDTs. The study will be conducted over approximately 18 months covering two high transmission seasons and the intervening dry season. The recruitment strategy aims to ensure that overall transmission and force of infection is not affected so we are able to continuously evaluate the impact of interventions in the context of on-going intense malaria transmission. The main objectives of the study are to determine the impact of enhanced CCM and MSAT on the prevalence and density of parasitemia and gametocytemia and the transmissibility of infections. This will be achieved by molecular detection of infections in all study participants during start and end season cross-sectional surveys and routine sampling of malaria positive individuals to assess their infectiousness to mosquitoes.

Ethics and dissemination

The study has been approved by the relevant institutional ethics committees in Burkina Faso and the UK. Findings will be reported through local community meetings, peer-reviewed publications and conference presentations.

Trial registration number

ClinicalTrials.gov identifier: NCT03705624

For peer review only

For peer review only - http://bmjopen.bmj.com/site/about/guidelines.xhtml

STRENGTHS AND LIMITATIONS OF THIS STUDY:

- This study evaluates for the first time the impact of enhanced community case management and monthly screening and treatment on the transmissibility of malaria from endemic populations to mosquitoes.
- The detailed examination of malaria infections, gametocyte production and infectivity to mosquitoes across two peak transmission seasons and the intervening low transmission season provides uniquely detailed information on the dynamics of parasite carriage and infectivity.
- Intensive monitoring of mosquito exposure in combination with repeated assessments of parasite carriage in the human population allows detailed assessments of the force of malaria infection at different time-points during the peak and low transmission season.
- A limitation of this current study is that findings from this setting where malaria transmission intensity is high, may not translate to areas of lower transmission intensity where the tested approaches may be used in malaria elimination efforts.
- The study specifically aims not to impact transmission or maximize the community effect of the interventions. Rather, the study aims to quantify the impact of enhanced community case management and monthly screening and treatment on the trajectory of infections experienced by the participating individuals, gametocyte production in these infections and transmissibility to mosquitoes.

KEY WORDS:

Malaria, transmission, direct membrane feeding assays, gametocyte, asymptomatic infections

For peer review only

INTRODUCTION:

Malaria continues to be a major public health problem in sub Saharan Africa. In 2017, there were an estimated 435,000 deaths from malaria globally, 93% in sub Saharan Africa (1). Despite this sobering figure and indications that no measurable reduction in global malaria cases has been achieved since 2015 (1), many countries had experienced a considerable decline in malaria transmission prior to this (2, 3) meaning malaria elimination and eradication are back on the agenda of the international research community and policy makers (4, 5). To achieve this, interventions that specifically aim to reduce transmission of malaria parasites are high on the priority list of the research agenda for malaria eradication (6, 7). Transmission of malaria begins with the formation of sexual-stage malaria parasites — the gametocytes. In *P. falciparum* infection, gametocytes develop and mature in the bone marrow and first appear in the circulation 10-12 days after asexual parasites are detected (8). Upon release in circulation, male and female gametocytes may persist for several weeks after asexual parasites have been cleared. In chronic infections there is persistent (but fluctuating) production of gametocytes from their asexual progenitors (9). Once ingested by a blood feeding female *Anopheles* mosquito, male and female gametocytes activate into gametes that fertilize and ultimately render the mosquito infectious to humans. Gametocytes are present not only in symptomatic malaria cases but also in infections not accompanied by symptoms that are severe enough to elicit treatment-seeking behaviour — so-called 'asymptomatic infections' (10). Since these asymptomatic infections represent a large proportion of all infections present in malaria-endemic settings (11, 12), asymptomatic parasite carriage may be a major component of the human infectious reservoir for malaria (13-15). Therefore detecting and treating these infections could be a valuable approach for reducing transmission. It is unclear how many asymptomatic infections start off as symptomatic infections and could potentially be detected and treated by enhanced community case management (CCM). With CCM, access to early diagnosis and treatment is improved by community based malaria diagnosis. CCM may involve the deployment of malaria posts that improve access to care while still relying on passive detection of infection or, in an enhanced format, may involve active screening for fever (16, 17). In an optimistic scenario, CCM could be used to prevent the majority of infectious days by abrogating infections before they become transmissible to mosquitoes. Alternatively, many incident infections may never elicit symptoms and would therefore remain undetected even during CCM with regular clinical examinations. In this situation, active screening approaches would be needed to identify asymptomatic infections for drug-based targeting to prevent or interrupt the production of infectious gametocytes. Mass screening and treatment (MSAT) approaches aim to detect asymptomatic infections by screening populations regardless of symptoms. Their ability to detect all infections depends on the diagnostic used (13) and in high endemic settings the impact of

For peer review only - http://bmjopen.bmj.com/site/about/guidelines.xhtml

MSAT may be transient (18). The number of infections acquired and the likelihood that infections give rise to clinical symptoms may be influenced by spatial and temporal variations in malaria exposure (19, 20). Thus, detailed entomological assessments may uncover small-scale variations in mosquito exposure that play a key role in determining the detectability and transmissibility of infections in a given area (21).

In this study, we evaluate the impact of two intensified diagnostic and treatment strategies on the human infectious reservoir of malaria, and compare these to the current standard of care. These diagnostic and treatment strategies aim to i) detect and treat symptomatic infections earlier and more comprehensively by enhanced community case management (CCM; weekly active screening for fever); and ii) detect and treat asymptomatic malaria infections by monthly screening with point-of-care rapid diagnostic tests (MSAT).

For peer review only

METHODS AND ANALYSIS:

Study design

This study is a cluster-randomised trial to determine the impact of two interventions on parasite carriage, gametocyte carriage and infectiousness to mosquitoes. A total of 180 compounds (consisting of 3-6 enrolled participants) will be randomised to one of three study arms (60 compounds per arm): arm 1 - current standard of care with passively monitored malaria infections; arm 2 - standard of care plus enhanced community case management (CCM); or arm 3 - standard of care and enhanced CCM, plus monthly screening and treatment (MSAT), as detailed in Table 1. The study will take place over approximately 18 months covering two high transmission seasons and the low transmission season (dry season) in between. Throughout the study, entomological monitoring will take place to evaluate the local mosquito population and potential exposure to infected mosquitoes.

Arm 1 (control): Standard of care
• Standard of care with passively monitored malaria incidence at local health facilities. Health facilities will be equipped with research staff and adequate supplies to ensure malaria diagnosis and treatment with the first line antimalarial artemether-lumefantrine (AL) according to national guidelines.<br>• Seasonal Malaria Chemoprevention in children <5 years of age implemented by the national malaria control programme (monthly treatment with sulfadoxine-pyrimethamine + amodiaquine from July to October).
**Arm 2 (intervention): CCM**
• Standard of care (as in arm 1)<br>• Seasonal Malaria Chemoprevention (as in arm 1)<br>• Enhanced Community Case Management for malaria (CCM) involving weekly active screening for fever using a research-grade thermometer by a trained health worker. A measured temperature ≥37.5°C or reported fever in the last 24 hours will prompt screening with a conventional rapid diagnostic test (RDT). RDT positive individuals will be treated with AL according to national guidelines
**Arm 3 (intervention): CCM+MSAT**
• Standard of care (as in arm 1 and 2)<br>• Seasonal Malaria Chemoprevention (as in arm 1 and 2)<br>• Enhanced CCM (as in arm 2)<br>• Monthly Screening and Treatment (MSAT) regardless of symptoms with a conventional RDT. Screening will be performed by research staff with 25-35 days between screening rounds; RDT positive individuals will be treated with AL according to national guidelines.

Table 1. Study arms

Gradual implementation of interventions

The aim of the study is to determine the ability of the interventions to detect malaria infections, and the transmission potential of these infections. To maximize coverage and ensure good adherence to intervention protocols the study will be implemented in two phases (Figure 1). Phase 1 will initiate prior to the first transmission season with 30 compounds enrolled per study arm. Phase 2 will initiate at the end of the first transmission season with an additional 30 compounds enrolled per study arm.

For peer review only - http://bmjopen.bmj.com/site/about/guidelines.xhtml

Parasitology data and mosquito infection data will be compiled and analysed before the start of the second transmission season (~May 2019), at which time sample size and study procedures may be amended upon ethical approval.

Study setting

This study will be conducted within the Saponé health and demographic surveillance system (HDSS) area, located 45 kilometres Southwest of Ouagadougou, Burkina Faso. This HDSS is part of the INDEPTH Network (a global network of health and demographic surveillance systems that provide a more complete picture of the health status of communities in low and middle income countries) and covers a total population of 85,000 living in 10,841 compounds. Compounds are geo-referenced and a census of the population is conducted annually. The population is served by 23 local health facilities and a district hospital. This is a region of intense and highly seasonal malaria transmission (predominanatly *P. falciparum*) with peak transmission from approximately June to October. Asexual parasite prevalence by microscopy in this population is ~50% in children below 15 years of age (14) and studies in this and other malaria-endemic settings in Burkina Faso indicate that ~80% of all asexual parasite carriers have concurrent gametocytes at highly variable densities (14, 22).

Enrolment and randomization

The unit of randomisation in this study is a compound. Per compound a minimum of one and maximum of two members will be enrolled from each of the three age groups (under 5 years of age, 5 – 15 years of age, and over 15 years of age), so that each compound will provide 3-6 enrolled participants. All participants must be willing to use the designated local health facilities, and to accommodate indoor and outdoor mosquito sampling for the study duration. Inclusion and exclusion criteria for individual study participants are listed in Table 2. From the 2017 census data, an estimated 4,700 compounds in the Saponé HDSS contained the required number of participants in the each age category and are in principle eligible. Up to date census data will be used prior to the study to select eligible compounds for enrolment in the Sapone Marche and Pissy local health areas (Figure 2). 180 compounds will be enrolled and will be divided into 60 groups of 3 based on proximity. Within each group, one compound will be randomly allocated to each of the three study arms by a computer generated algorithm. To enable the impact of the interventions to be continually evaluated throughout the study period, the study is designed so that overall transmission intensity is not influenced. To ensure the force of infection remains high ≤50% of all compounds in a given area will be enrolled in the study. This will be achieved by combining each group of 3 enrolled compounds with 3 non-enrolled adjacent compounds to form a total of 60 groups (Figure 3). Simulations predict

For peer review only

For peer review only - http://bmjopen.bmj.com/site/about/guidelines.xhtml

that the interventions will have very small impact on overall transmission in the study area relative to the current transmission intensity (Figure 4).

Inclusion criteria	
i.	Participants should be permanent residents of the compound
ii.	Participants should be willing to participate in repeated assessments of health and infection status and willing to donate a maximum of 37mL of blood (children <10 years of age) or 52mL of blood (older individuals) during an 18-month period
**Exclusion criteria**	
i.	Any (chronic) illness that would affect study participation
ii.	Pre-existing severe chronic health conditions
iii.	Current participation in malaria vaccine trials or participation in such trials in the last 2 years
iv.	History of intolerance to artemether-lumefantrine

Table 2. Inclusion and exclusion criteria for enrolled participants

Procedures

Study visits

At the start and end of each season, a cross-sectional survey will be performed concurrently in all enrolled compounds (Figure S1). These surveys will also include adults that are present in enrolled compounds but not participating in the interventions; additional informed consent will be obtained for this. In these surveys all individuals will have their axillary temperature measured and a finger prick blood sample taken for molecular assessment of gametocyte and parasite carriage (as detailed in Table 3.). In case of measured fever, the same finger prick blood sample will be used for malaria diagnosis by conventional rapid diagnostic test (RDT: First Response Malaria Ag. pLDH/HRP2, specific for *P. f* and/or *P. v, P. o* and *P. m*) and treatment will be offered as per national guidelines. Routine surveys will be conducted throughout the wet and dry seasons to assess parasite carriage, gametocyte carriage and infectivity to mosquitoes. These surveys will be performed on a subset of compounds each week on a rolling basis, so that each compound will visited every 10 weeks. Finger prick blood samples will be taken and processed as detailed in Table 3; one whole blood aliquot will be processed immediately for molecular detection of *P. falciparum* parasitemia by quantitative PCR (qPCR) targeting varATS (23). qPCR positive individuals (i.e. those with parasites, not necessarily gametocytes) will be invited to donate blood for mosquito feeding assays. The maximum number of feeding assays per day will be dependent on logistical feasibility. Asymptomatic infections that are detected by PCR will not be treated if unaccompanied by malaria symptoms, in line with national treatment guidelines. Routine surveys will continue throughout the dry season to allow a detailed assessment of the number of newly acquired infections when minimal exposure to infected mosquito bites is assumed.

For peer review only

For peer review only - http://bmjopen.bmj.com/site/about/guidelines.xhtml

In all three arms, clinical malaria episodes will be passively monitored at the local health facilities. Participants will be identified using study ID cards; for suspected malaria cases temperature and clinical history will be recorded. Fingerprick blood samples will be taken as detailed in Table 3. Malaria diagnosis will be performed by RDT and infected subjects will be treated with AL. For infections detected by community health workers during enhanced CCM (arm 2 and 3) or MSAT (arm 3) visits, the same procedure is used and samples are collected as detailed in Table 3. For RDT or microscopy positive infections detected at local health facilities or during the CCM or MSAT visits, when logistically feasible the individuals will be invited to donate a blood sample for mosquito feeding assays prior to treatment. For all mosquito feeding assays blood samples will be collected and transported in a portable incubator or thermos flask to the insectary for direct membrane feeding assays (DMFA).

Sample type/Procedure	Cross-sectional survey	Routine survey	Passive case detection	CCM	MSAT	DMFAs
Axillary temperature and clinical assessment	X	X	X	X	X	
Dried blood spot (DBS)	X	X	X	X*	X	
Whole blood in RNA protect	X	X	X	X*	X	X
Whole blood for DNA	X	X	X	X*	X	X
Rapid diagnostic test (RDT)	X*		X	X*	X	
Highly sensitive RDT (hsRDT)						X
Plasma						X
Blood smear			$X^{\#}$	$X^{\#}$	$X^{\#}$	X

Table 3. Sampling schedule: Table indicates the samples collected at each participant survey/visit. X* = sampling only takes place if subject has fever or history of fever in last 24 h; $X^{\#}$ = sampling only takes place if subject has history of antimalarial treatment in previous 3 weeks for microscopic confirmation of malaria infection; CCM = enhanced community case management visit; MSAT = monthly screening and treatment visit; DMFA = direct membrane feeding assay.

Entomology

Detailed assessments of mosquito exposure will be performed at the compound level. Mosquito exposure will primarily be determined using a CDC miniature light trap (Model 512; John W. Hock Company, Gainesville, Florida, USA) placed inside one of the sleeping spaces close to participants sleeping under a net. Sampling will be performed in every compound once every 14 days, throughout the study. To explore outdoor biting and enable more sensitive sampling of sparse mosquito

For peer review only

For peer review only - http://bmjopen.bmj.com/site/about/guidelines.xhtml

populations, Human Landing Catches (HLC) will be performed indoors and outdoors from dusk until dawn for a subset of compounds (24). Due to the low densities of mosquitoes present in the dry season, additional strategies will be used to optimise the potential for mosquito monitoring during that period. Dry season pyrethrum spray catches will be conducted in a selection of non-enrolled compounds to collect indoor resting mosquitoes. These compounds will be sprayed with the insecticide aerosol Kaltox® (a mixture of pyrethroids and carbamate containing 0.27 % Allethrin, 0.2 % Tetramethrin, 0.17 % Permethrin, and 0.68 % Propoxur) for 30-45 seconds; dead and immobilized mosquitoes will be collected after 10 minutes. In addition, in the absence of water body larval habitats in the dry season an active search of refugia and resting trap collections may be performed close to study compounds. At all collections, factors such as housing type and presence of animals will be recorded and associations with mosquito exposure will be examined. Throughout the study, presence, size and location of water bodies will be periodically assessed.

Collected mosquitoes will be sorted and counted by sex and species using morphological characteristics, and classified according to blood feeding status and parity (unfed, blood-fed, semi-gravid and gravid) by microscopy. A subset of mosquitoes will be preserved for further molecular analysis (25, 26). Age structure of the mosquito population will be determined by examination of tracheal distension within the ovaries (27). Infection status of Anopheles caught in CDC traps will be determined using circumsporozoite ELISA (CSP-ELISA) (28, 29) and or PCR (30, 31).

Laboratory assessments

Direct membrane feeding assays (DMFA) will be performed to determine infectivity to locally reared female *Anopleles coluzzii* mosquitoes, as described previously (14). Whole blood collected in lithium heparin tubes will be offered in duplicate to 50-60 starved mosquitoes via an artificial membrane feeder (400-500 µl per feeder). Fully fed mosquitoes will be kept at 27-29°C and maintained on a sucrose diet for approximately one week (6 – 8 days). Mosquito infection will be determined by dissection and assessment for the presence and density of oocysts by two independent microscopists. Infected midguts will be stored for molecular confirmation of infection and genotyping for a subset of guts (14).

Parasite DNA and RNA will be extracted from whole blood samples collected in EDTA tubes. Parasitemia will be assessed using varATS qPCR (23) and gametocyte density and sex ratio will be assessed using established qRT-PCR protocols (32). Whole blood samples will be evaluated by HS-RDTs and presence and quantification of HRP2 will be also determined using the bead-based Luminex® (Austin, TX) platform (33). Multiplicity of infection will be determined in a selection of samples using MSP-genotyping (34) or equivalent techniques.

For peer review only - http://bmjopen.bmj.com/site/about/guidelines.xhtml

Red blood cell polymorphisms that are associated with the likelihood of developing blood-stage (clinical) malaria infection, the severity of malaria infection or gametocyte production (haemoglobin C, haemoglobin S, α-thalassaemia and glucose-6-dehydrogenase deficiency) (35) will be measured in baseline samples by multiplex luminex assay (36). In secondary analyses, antibody responses to parasite and gametocyte antigens will be determined by protein microarray (37) and multiplex bead-based serological assays (38) and associated with parasite carriage, clinical symptoms and infectivity. For a selection of samples functional transmission reducing immunity will be assessed by standard membrane feeding assay (SMFA) using plasma samples collected at the time of field-based DMFA (39).

Objectives/endpoints

The overall aim of the study is to determine the impact of the two diagnostic strategies on prevalence and transmissibility of infections. The hypothesis is that the parasite prevalence and density are reduced in arms 2 and 3 compared to the standard of care. More infections will be detected early and may therefore be abrogated before they develop into chronic asymptomatic infections with continuous gametocytemia. As such the primary endpoint is parasite prevalence and density in each arm by molecular detection in the end of study cross-sectional survey. Secondary objectives and endpoints seek to evaluate the impact of the interventions after each season, and to define the contribution and infectivity of gametocytes in these infections, as detailed in Table 4. Exploratory objectives aim to understand the detectability and transmissibility of infections, by exploring i) relationships between parasitemia and transmission, and ii) associations between infectivity and host characteristics, parasite characteristics, or mosquito exposure (Table 4).

For peer review only - http://bmjopen.bmj.com/site/about/guidelines.xhtml

Primary objective/endpoint
1. Parasite prevalence and density between arms by molecular detection at the end of study cross-sectional survey. [Time Frame: Month 18 (end of second transmission season; January-February 2020)]
**Secondary objectives/endpoints**
1. Parasite prevalence and density by molecular detection at the end of year 1 cross-sectional survey. [Time Frame: Month 6 (end of first transmission season; January-February 2019)]
2. Parasite prevalence and density by molecular detection at the end of the dry season cross-sectional survey. [Time Frame: Month 12 (prior to second transmission season; June 2019)]
3. Gametocyte prevalence and or density by molecular methods at the end of study cross-sectional survey. [Time Frame: Month 18 (end of second transmission season; January-February 2020)]
4. Gametocyte prevalence and or density by molecular methods at the end of year 1 cross-sectional survey. [Time Frame: Month 6 (end of first transmission season; January-February 2019)]
5. Gametocyte prevalence and or density by molecular methods at the end of the dry season cross-sectional. [Time Frame: Month 12 (prior to second transmission season; June 2019)]
6. Gametocyte prevalence and or density by molecular methods among *P. falciparum* infections during all visits in the study. [Time Frame: Throughout study, an average of 18 months]
7. The number of incident infections/clinical incidence detected during CCM, MSAT and passive case detection. [Time Frame: Throughout study, an average of 18 months]
8. Infectivity to mosquitoes of *P. falciparum* infections [Time Frame: Throughout study, an average of 18 months]
**Exploratory objectives/endpoints:**
1. The detectability of infections by highly sensitive rapid diagnostic tests. [Time Frame: Throughout study, an average of 18 months]
2. The relationship between the proportion of infected mosquitoes and gametocyte density. [Time Frame: Throughout study, an average of 18 months]
3. The impact of infection characteristics on the transmissibility of infections to mosquitoes [Time Frame: Throughout study, an average of 18 months]
4. The impact of human host characteristics on the transmissibility of infections to mosquitoes [Time Frame: Throughout study, an average of 18 months]
5. Association between total parasite density and gametocyte density [Time Frame: Throughout study, an average of 18 months]
6. Malaria transmission potential based on measured gametocyte densities and the association between gametocyte density and mosquito infection rates [Time Frame: Throughout study, an average of 18 months]
7. Number of acquired clones based on genotyping [Time Frame: Throughout study, an average of 18 months]
8. The duration of infections in the dry season [Time Frame: Month 6-12, between end of season survey January/February 2019 and end of dry season survey June 2019]
9. To quantify mosquito exposure in relation to incident infections and transmission opportunities [Time Frame: Throughout study, an average of 18 months]

Table 4. Objectives and endpoints

CCM = enhanced community case management involving active weekly fever screening; MSAT = Monthly screening and treatment

For peer review only

For peer review only - http://bmjopen.bmj.com/site/about/guidelines.xhtml

Data analysis

For primary and secondary endpoints, parasite and gametocyte prevalence will be compared between arms using multivariate mixed effect logistic regression models; parasite and gametocyte density will be assessed using multivariate mixed effect linear regression models on log transformed values. The number of incident infections will be assessed by Poisson regression and compared between arms. Proportion of infectious participants and proportion of infected mosquitoes will be assessed by logistic regression models. Analyses will take account of correlation of outcomes at the compound level and the matched randomisation scheme. There may be issues with convergence in mixed effect models given the small number of subjects per compound. In this case analysis based on compound level summaries will be performed. For exploratory endpoints, detected infections will be analysed in relation to the time and duration of infection, parasite and gametocyte prevalence and density, symptoms, complexity of infection, host characteristics, mosquito exposure and infectiousness to mosquitoes. Parasite density will be related to detectability by (ultra-sensitive) rapid diagnostic tests. The association between gametocyte density and mosquito infection prevalence and intensity will be determined using an established Bayesian hierarchical model that incorporates the density of male gametocytes and gametocyte sex ratio (40). In exploratory endpoints, model fitting will be performed for different strata of infection characteristics (e.g. complexity of infection, concurrent asexual parasite density, and season) and host characteristics (e.g. anti-gametocyte immune responses, age). Mosquito exposure by indoor and outdoor trapping methods will be associated with the likelihoods of incident infections and opportunities to transmit infections using comprehensive malaria transmission models (41).

Sample size calculations and predicted impact of interventions

The hypothesis of the current study is that the parasite prevalence is considerably reduced in arms 2 and 3 compared to the standard of care. Assuming a conservative parasite prevalence of 40% in the control arm, the current sample size should be sufficient to detect a 2-fold reduction in parasite prevalence with >99% power. This sample size will also allow us to detect the anticipated difference in the proportion of infections that are gametocyte-positive at the moment of detection (secondary objectives 3-5). We assume that ≥80% of all infections detected in cross-sectional surveys (i.e. chronic asymptomatic infections) concurrently carry gametocytes (14). We expect that infections that are detected by CCM or MSAT visits in arms 2 and 3 are of shorter duration and have lower gametocyte prevalence and density (15). If we assume ≤50% gametocyte prevalence among these CCM/MSAT-detected infections, monitoring 100 survey-detected and 100 CCM/MSAT-detected infections will result in 99% power to detect this difference (the calculations assumed a coefficient of variation of 0.5 and a significance level of 0.05).

For peer review only - http://bmjopen.bmj.com/site/about/guidelines.xhtml

Simulations predict that enhanced CCM will reduce asexual parasite carriage in arm 2 by 70%, 60%, and 30% in children under 5, 5-15 year olds, and adults > 15 years, respectively. The addition of MSAT in arm 3 is predicted to have minimal impact in all age groups (Figure 5). Simulations were carried out in EMOD v2.15 (42, 43) an agent-based model of malaria transmission using within-host infection, immunity, and infectivity dynamics (41, 44) calibrated to field data (45). The study site census was gridded into 1km grid cells with total human population of 7,800 individuals and birth and death rates of 39 per 1,000 per year, resulting in 17% of population being under 5 and 25% between 5 and 15. Within a grid cell, biting rate is surface area dependent but otherwise homogeneous. Humans moved between grid cells at a rate of one trip per person per year, and each trip lasted on average two days. Vectors did not move between grid cells. Malaria dynamics are simulated in a highly seasonal setting with annual EIR of 150 in the presence of ITNs. Seasonality was based on entomology data collected in 2011. The vector population was modeled as *An. gambiae* mosquitoes with 65% anthropophily and 90% indoor feeding. All grid cells had the same seasonality and transmission intensity. For the simulations, the number of individuals per arm were ~165 during phase 1 until January 2019 when numbers increased by ~200 individuals per arm for phase 2. Means of 500 stochastic realizations are shown. Simulated interventions are as follows:

1. **SMC –** Administration of a curative drug with prophylactic protection for 30 days to children < 5 years. Four rounds of SMC carried out each year starting June 30 with 30 days between rounds. Coverage was set at 80% per round and independent between rounds.

2. **ITNs – Estimated age dependent distribution;** 0-5y = 67%, 5-10y = 74%, 10-15y = 68%, 15-20y = 65%, and >20y = 71%.

3. **Passive case detection and health seeking behaviour –** AL administered to uncomplicated and severe malaria cases with age-dependent distance-based health seeking. Distances to the two study health facilities in Sapone and Pissy were calculated from the center of each grid cell with exponential half-distance in coverage of 10 km. Maximum coverage was 50% for children under 5, 30% for individuals over 5, and 80% for severe cases. Patients received treatment within 3 days of onset of clinical symptoms.

4. **Enhanced community case management (CCM) in arms 2 and 3 –** Modeled as continuous weekly active screening where fevers of 37.5°C or higher receive AL, beginning June 30, 2018 with 100% coverage.

For peer review only

For peer review only - http://bmjopen.bmj.com/site/about/guidelines.xhtml

5. **MSAT with RDT in arm 3 –** Modeled as continuous monthly RDT testing (diagnostic sensitivity 40 asexual parasites/µL), with RDT positive individuals receiving AL, beginning June 30, 2018 with 100% coverage.

Patient and public involvement

Patients and public were not involved in the design of this study. The findings of the study will be shared with the community via the local opinion leaders. The project is followed by a community meeting to present some of the preliminary findings. Disease occurrence data will be shared periodically with the Ministry of Health and we will work closely with the National Malaria Control Programme. The results may also be shared in conferences, seminars, reports, theses, and peer-reviewed publications.

ETHICS AND DISSEMINATION:

Ethical considerations

The purpose of the study and practical consequences of participation will be explained during both community meetings and visits to each household by experienced nurses. Objectives and procedures will be explained verbally and given in printed form in French or a local language. All compound members will be screened by a GCP-trained clinician for enrolment criteria, signs of acute disease, and individual informed consent obtained (consent obtained from parent or legal guardian in the case of minors). Malaria infections will not be cleared prior to inclusion in the study but this may be considered later based on results of the first season. Payment will not be given for participation but instead participants will receive financial compensation for travel and time. In addition, each participant enrolled in the study will receive a free long-lasting insecticide treated bed net and treatment for malaria and other common illnesses free of charge. The blood draw volumes will be minimized: a maximum of 6 mL of blood will be taken at any single time-point and the total volume of blood taken during a 6 month period will be lower than 37 mL for individuals enrolled in arms 3, or lower than 22 mL for those in arms 1 and 2. Both the cumulative blood draw volume and maximum blood volume taken in a single sample are within the limits considered as physiological “minimal risk” (46). No experimental medication is used in this study; AL is approved in country as first-line antimalarial treatment. Study identification numbers will be used on all paper and electronic questionnaires. Some questionnaires will be programmed using OpenDataKit with data entered into password-secured, encrypted tablets. Data will be uploaded daily to an encrypted cloud-based sever. The study has been reviewed and approved by London School of Hygiene and Tropical Medicine (LSHTM) (Review number: 14724) and The Centre National de Recherche et de Formation sur le Paludisme institutional review board (IRB) (Deliberation N° 2018/000002/MS/SG/CNRFP/CIB) and

For peer review only

Burkina Faso national medical ethics committees. (Deliberation N° 2018-01-010). Any modifications to the protocol which may impact on the conduct of the study, subject safety or potential benefit of the subjects, including changes of study objectives, study design, study procedures, study population or sample size will require a formal amendment to the protocol. Such amendment will be approved by the Ethics Committees/IRB prior to implementation. The current protocol version is 2.0 approved November 2018. The full World Health Organization Trial Registration Data Set information is included in the supplementary Table S1. The trial was submitted to ClinicalTrials.gov (NCT03705624) prior to study initiation on the 19th July 2018. Delays in releasing the registration were related to minor textual changes required to adhere to ClincalTrials.gov formatting requirements and did not change the content of the submission, but resulted in first release online in October."

Trial oversight, monitoring and quality control

This study will comply with GCP guidelines and be conducted in accordance with the latest South Africa revision of the Declaration of Helsinki and local regulatory requirements. All study staff will receive a local workshop in Good Clinical Practice before the study starts. Since no experimental treatment is used in the study a Data Safety and Monitoring Committee will not be installed; a scientific steering committee will provide technical oversight and will assist with interpretation of data. LSHTM is the Research Sponsor, taking responsibility for the initiation and management of the trial clinical trial including trial monitoring. The LSHTM Research Governance and Integrity Office has reviewed, assessed and registered the trial and holds Public Liability ("negligent harm") and Clinical Trial ("non-negligent harm") insurance policies which apply to this trial.

Periodically, a subset of compounds will be randomly selected from each arm to be visited by the quality control team to monitor study implementation adherence and unintended effects of study interventions or conduct. Quality control for mosquito feeding assays includes regular enrolment of microscopy-positive gametocyte carriers as controls. For this, children aged 10-15 years from non-participating compounds will be screened for the presence of gametocytes by microscopy and up to 5 gametocyte carriers will be enrolled every 6 weeks. Specific informed consent will be provided for this.

Dissemination

Findings of the study will be shared with the community via local opinion leaders and community meetings. Results may also be shared through conferences, seminars, reports, theses, and peer-reviewed publications; disease occurrence data and study outcomes will be shared with the Ministry of Health. Data will be published in an online digital repository.

For peer review only - http://bmjopen.bmj.com/site/about/guidelines.xhtml

AKNOWLEDGMENTS:

The authors would like to thank the following individuals for their contributions to the study protocol development: Bronner Gonçalves, Umberto D'Alessandro, Edward Wenger, Lorenz von Seidlein & Tovi Lehman. Patient advisors were not required as part of the design of this study.

AUTHORS CONTRIBUTIONS:

AT, TB, CD and KC developed and designed the study. WMG, AO, SA, IS, AN, PS, JG, JB. MO AD and WS contributed to study design. All authors contributed to protocol development. KC, AO, CD, TB and AT wrote the manuscript. All authors reviewed and approved the manuscript final draft.

FUNDING STATEMENT:

This study is funded by Bill and Melinda Gates Foundation grant (OPP1173572). KC and TB are also supported by a fellowship from the European Research Council (ERC-2014-StG639776). AT is supported by an award from UK Medical Research Council (MRC) and the UK Department for International Development (DFID) under the MRC/DFID Concordat agreement (MR/P02016X/1)

COMPETING INTEREST STATEMENT:

All authors declare no competing interest.

ROLE OF FUNDING SOURCE

The funders, Bill &Melinda Gates Foundation, were involved in study design, but had no role in data collection, analysis, interpretation, or reporting.

For peer review only

REFERENCES:

1. World Health Organization (WHO). World Malaria Report 2018. https://www.who.int/malaria/publications/world-malaria-report-2018/en/: .
2. Bhattarai A, Ali AS, Kachur SP, et al. Impact of artemisinin-based combination therapy and insecticide-treated nets on malaria burden in Zanzibar. *PLoSMed*. 2007;4(11):e309.
3. Barnes KI, Durrheim DN, Little F, et al. Effect of artemether-lumefantrine policy and improved vector control on malaria burden in KwaZulu-Natal, South Africa. *PLoSMed*. 2005;2(11):e330.
4. Alonso PL, Brown G, Arevalo-Herrera M, et al. A research agenda to underpin malaria eradication. *PLoS Med*. 2011;8(1):e1000406.
5. Greenwood BM. Control to elimination: implications for malaria research. *Trends Parasitol*. 2008;24(10):449-54.
6. MalERA Consultative Group on Drugs. A research agenda for malaria eradication: drugs. *PLoS medicine*. 2011;8(1):e1000402.
7. MalERA Consultative Group on Vaccines. A research agenda for malaria eradication: vaccines. *PLoS medicine*. 2011;8(1):e1000398.
8. Eichner M, Diebner HH, Molineaux L, et al. Genesis, sequestration and survival of Plasmodium falciparum gametocytes: parameter estimates from fitting a model to malaria therapy data. *Trans R Soc Trop Med Hyg*. 2001;95.
9. Johnston GL, Smith DL, Fidock DA. Malaria's missing number: calculating the human component of R0 by a within-host mechanistic model of Plasmodium falciparum infection and transmission. *PLoS Comput Biol*. 2013;9(4):e1003025.
10. Bousema T, Drakeley C. Epidemiology and infectivity of Plasmodium falciparum and Plasmodium vivax gametocytes in relation to malaria control and elimination. *Clinical microbiology reviews*. 2011;24(2):377-410.
11. Imwong M, Stepniewska K, Tripura R, et al. Numerical Distributions of Parasite Densities During Asymptomatic Malaria. *J Infect Dis*. 2016;213(8):1322-9.
12. Bousema T, Okell L, Felger I, et al. Asymptomatic malaria infections: detectability, transmissibility and public health relevance. *Nature reviews Microbiology*. 2014;12(12):833-40.
13. Slater HC, Ross A, Ouedraogo AL, et al. Assessing the impact of next-generation rapid diagnostic tests on Plasmodium falciparum malaria elimination strategies. *Nature*. 2015;528(7580):S94-101.
14. Goncalves BP, Kapulu MC, Sawa P, et al. Examining the human infectious reservoir for Plasmodium falciparum malaria in areas of differing transmission intensity. *Nat Commun*. 2017;8(1):1133.
15. Tadesse FG, Slater HC, Chali W, et al. The relative contribution of symptomatic and asymptomatic Plasmodium vivax and Plasmodium falciparum infections to the infectious reservoir in a low-endemic setting in Ethiopia. *Clin Infect Dis*. 2018.
16. Ndiaye JLA, Ndiaye Y, Ba MS, et al. Seasonal malaria chemoprevention combined with community case management of malaria in children under 10 years of age, over 5 months, in south-east Senegal: A cluster-randomised trial. *PLoS medicine*. 2019;16(3):e1002762.
17. Landier J, Parker DM, Thu AM, et al. Effect of generalised access to early diagnosis and treatment and targeted mass drug administration on Plasmodium falciparum malaria in Eastern Myanmar: an observational study of a regional elimination programme. *Lancet*. 2018;391(10133):1916-26.
18. Tiono AB, Ouedraogo A, Ogutu B, et al. A controlled, parallel, cluster-randomized trial of community-wide screening and treatment of asymptomatic carriers of Plasmodium falciparum in Burkina Faso. *Malar J*. 2013;12:79.
19. Bousema T, Griffin JT, Sauerwein RW, et al. Hitting hotspots: spatial targeting of malaria for control and elimination. *PLoS medicine*. 2012;9(1):e1001165.

For peer review only - http://bmjopen.bmj.com/site/about/guidelines.xhtml

20. Bejon P, Williams TN, Liljander A, et al. Stable and unstable malaria hotspots in longitudinal cohort studies in Kenya. *PLoS medicine*. 2010;7(7):e1000304.
21. Rodriguez-Barraquer I, Arinaitwe E, Jagannathan P, et al. Quantification of anti-parasite and anti-disease immunity to malaria as a function of age and exposure. *eLife*. 2018;7.
22. Ouedraogo AL, Schneider P, de Kruijf M, et al. Age-dependent distribution of Plasmodium falciparum gametocytes quantified by Pfs25 real-time QT-NASBA in a cross-sectional study in Burkina Faso. *AmJTropMedHyg*. 2007;76(4):626-30.
23. Hofmann N, Mwingira F, Shekalaghe S, et al. Ultra-sensitive detection of Plasmodium falciparum by amplification of multi-copy subtelomeric targets. *PLoS medicine*. 2015;12(3):e1001788.
24. Riehle MM, Guelbeogo WM, Gneme A, et al. A cryptic subgroup of Anopheles gambiae is highly susceptible to human malaria parasites. *Science*. 2011;331(6017):596-8.
25. Koekemoer LL, Kamau L, Hunt RH, et al. A cocktail polymerase chain reaction assay to identify members of the Anopheles funestus (Diptera: Culicidae) group. *Am J Trop Med Hyg*. 2002;66(6):804-11.
26. Santolamazza F, Calzetta M, Etang J, et al. Distribution of knock-down resistance mutations in Anopheles gambiae molecular forms in west and west-central Africa. *Malar J*. 2008;7:74.
27. Detinova TS. Determination of the physiological age of Anopheles females by the changes in the tracheal system of the ovaries. *Med Parazitol (Mosk)*. 1945;14(2):45-9.
28. Wirtz RA, Burkot TR, Graves PM, et al. Field evaluation of enzyme-linked immunosorbent assays for Plasmodium falciparum and Plasmodium vivax sporozoites in mosquitoes (Diptera: Culicidae) from Papua New Guinea. *Journal of medical entomology*. 1987;24(4):433-7.
29. Wirtz RA, Duncan JF, Njelesani EK, et al. ELISA method for detecting Plasmodium falciparum circumsporozoite antibody. *Bulletin of the World Health Organization*. 1989;67(5):535-42.
30. Snounou G, Viriyakosol S, Zhu XP, et al. High sensitivity of detection of human malaria parasites by the use of nested polymerase chain reaction. *Molecular and biochemical parasitology*. 1993;61(2):315-20.
31. Graumans W, Tadesse FG, Andolina C, et al. Semi-high-throughput detection of Plasmodium falciparum and Plasmodium vivax oocysts in mosquitoes using bead-beating followed by circumsporozoite ELISA and quantitative PCR. *Malar J*. 2017;16(1):356.
32. Meerstein-Kessel L, Andolina C, Carrio E, et al. A multiplex assay for the sensitive detection and quantification of male and female Plasmodium falciparum gametocytes. *Malar J*. 2018;17(1):441.
33. Plucinski MM, Dimbu PR, Fortes F, et al. Post-Treatment HRP2 Clearance in Patients with Uncomplicated Plasmodium falciparum Malaria. *J Infect Dis*. 2017.
34. Felger I, Maire M, Bretscher MT, et al. The dynamics of natural Plasmodium falciparum infections. *PLoS One*. 2012;7(9):e45542.
35. Lopera-Mesa TM, Doumbia S, Konate D, et al. Effect of red blood cell variants on childhood malaria in Mali: a prospective cohort study. *Lancet Haematol*. 2015;2(4):e140-9.
36. Grignard L, Mair C, Curry J, et al. Bead-based assays to simultaneously detect multiple human inherited blood disorders associated with malaria. *Malar J*. 2019;18(1):14.
37. Stone WJR, Campo JJ, Ouedraogo AL, et al. Unravelling the immune signature of Plasmodium falciparum transmission-reducing immunity. *Nat Commun*. 2018;9(1):558.
38. Varela ML, Mbengue B, Basse A, et al. Optimization of a magnetic bead-based assay (MAGPIX((R))-Luminex) for immune surveillance of exposure to malaria using multiple Plasmodium antigens and sera from different endemic settings. *Malar J*. 2018;17(1):324.
39. Ponnudurai T, Lensen AH, Van Gemert GJ, et al. Infectivity of cultured Plasmodium falciparum gametocytes to mosquitoes. *Parasitology*. 1989;98 Pt 2:165-73.
40. Bradley J, Stone W, Da D, et al. Predicting the likelihood and intensity of mosquito infection from sex specific Plasmodium falciparum gametocyte density *eLife*. 2018;7: pii: e34463.
41. Selvaraj P, Wenger EA, Gerardin J. Seasonality and heterogeneity of malaria transmission determine success of interventions in high-endemic settings: a modeling study. *BMC infectious diseases*. 2018;18(1):413.

For peer review only - http://bmjopen.bmj.com/site/about/guidelines.xhtml

42. Eckhoff PA. A malaria transmission-directed model of mosquito life cycle and ecology. *Malar J*. 2011;10:303.
43. Bershteyn A, Gerardin J, Bridenbecker D, et al. Implementation and applications of EMOD, an individual-based multi-disease modeling platform. *Pathog Dis*. 2018.
44. Eckhoff P. P. falciparum infection durations and infectiousness are shaped by antigenic variation and innate and adaptive host immunity in a mathematical model. *PLoS One*. 2012;7(9):e44950.
45. Ouedraogo AL, Goncalves BP, Gneme A, et al. Dynamics of the Human Infectious Reservoir for Malaria Determined by Mosquito Feeding Assays and Ultrasensitive Malaria Diagnosis in Burkina Faso. *J Infect Dis*. 2016;213(1):90-9.
46. Howie SR. Blood sample volumes in child health research: review of safe limits. *Bulletin of the World Health Organization*. 2011;89(1):46-53.

FIGURES LEGENDS:

Figure 1. Study flow diagram

Compounds are enrolled into the study during 2 Phases of recruitment and randomised to the three study arms. Enhanced CCM = Community case management, MSAT = Monthly screening and treatment, LHF = local health facility.

Figure 2. Map of Sapone HDSS

Sapone Health and Demographic Surveillance System (HDSS) area with selected study area in highlighted in orange

Figure 3. Study enrolment strategy

Health and demographic surveillance system census data was used to select eligible compounds based on the number and age of occupants. Eligible compounds were enrolled and groups of 3 enrolled compounds were combined with 3 non-enrolled compounds, generating 60 groups. Within each group the 3 enrolled compounds were randomly assigned to each of the 3 study arms. This strategy is used to ensure 1) an even spread or compounds in each arm over the study area and, 2) that ≤50% of all compounds in the study area are enrolled in the study, so overall transmission intensity remains unaffected.

Figure 4. Interventions have minimal impact on overall transmission in the study area relative to transmission intensity

True prevalence of any malaria infection in simulations with INDIE interventions (red) and without (blue) is similar. Simulated INDIE activities begin in July 2018 and continue through the end of simulation. Lines indicate mean and shaded area the 95% observed interval across 25 stochastic realizations.

For peer review only

570 **Figure 5. Simulations predict impact of interventions in arms 2 and 3 across age groups**

571 Simulation of the impact of enhanced CCM (arm 2, green) and enhanced CCM+MSAT (arm 3, orange)
572 relative to no intervention (arm 1, purple) on parasite carriage in young children, school-age children,
573 and adults. Lines indicate mean and shaded area the 95% observed interval of prevalence by PCR
574 with sensitivity 4 parasites/μL across 500 stochastic realizations.

For peer review only

For peer review only - http://bmjopen.bmj.com/site/about/guidelines.xhtml

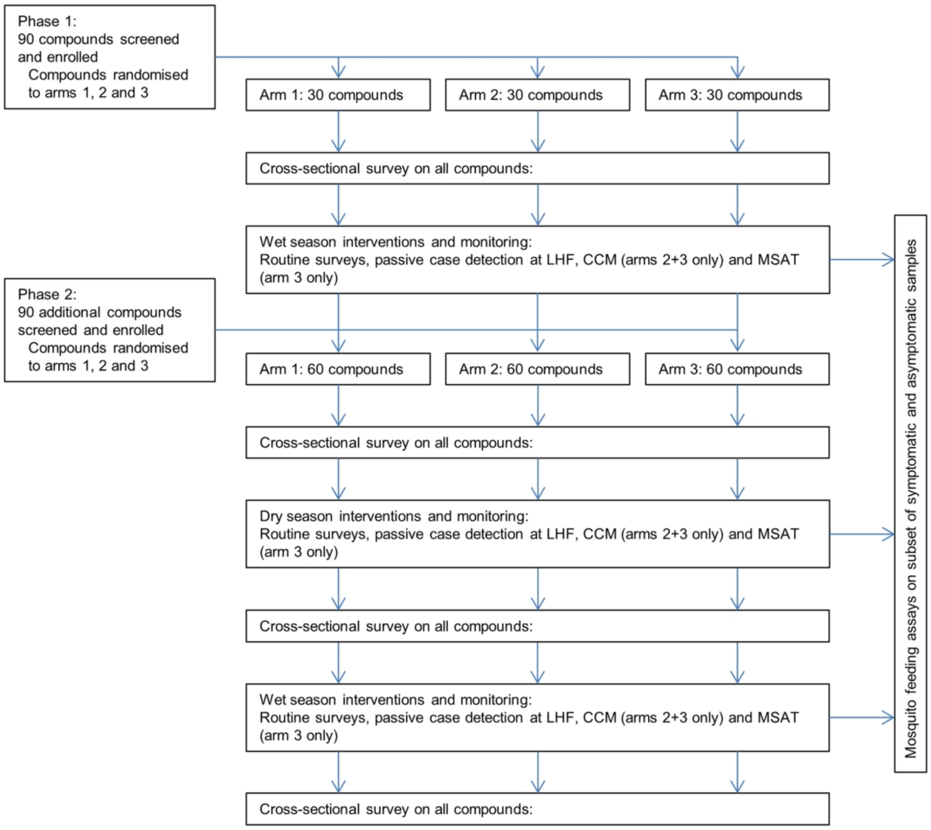

Figure 1

190x162mm (300 x 300 DPI)

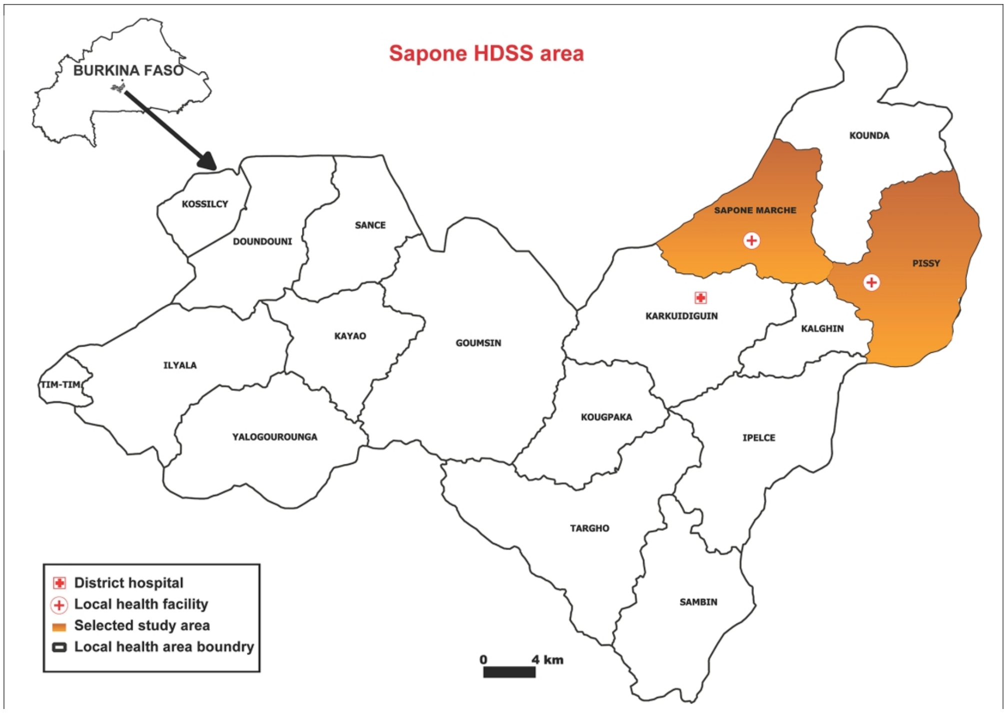
Sapone HDSS area
BURKINA FASO
KOSSILCY
DOUNDOUNI
SANCE
KOUNDA
SAPONE MARCHE
PISSY
KARKUIDIGUIN
KALGHIN
KAYAO
GOUMSIN
ILYALA
TIM-TIM
YALOGOUROUNGA
KOUGPAKA
IPELCE
TARGHO
SAMBIN
District hospital
Local health facility
Selected study area
Local health area boundry
0
4 km

Figure 2

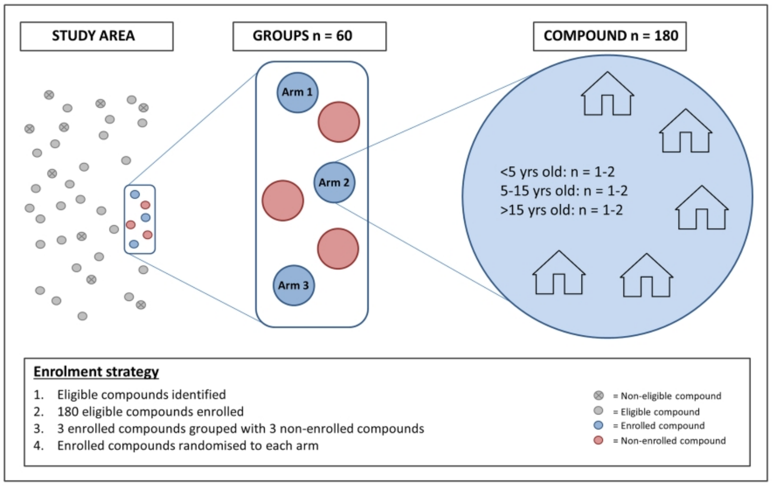

399x279mm (300 x 300 DPI)

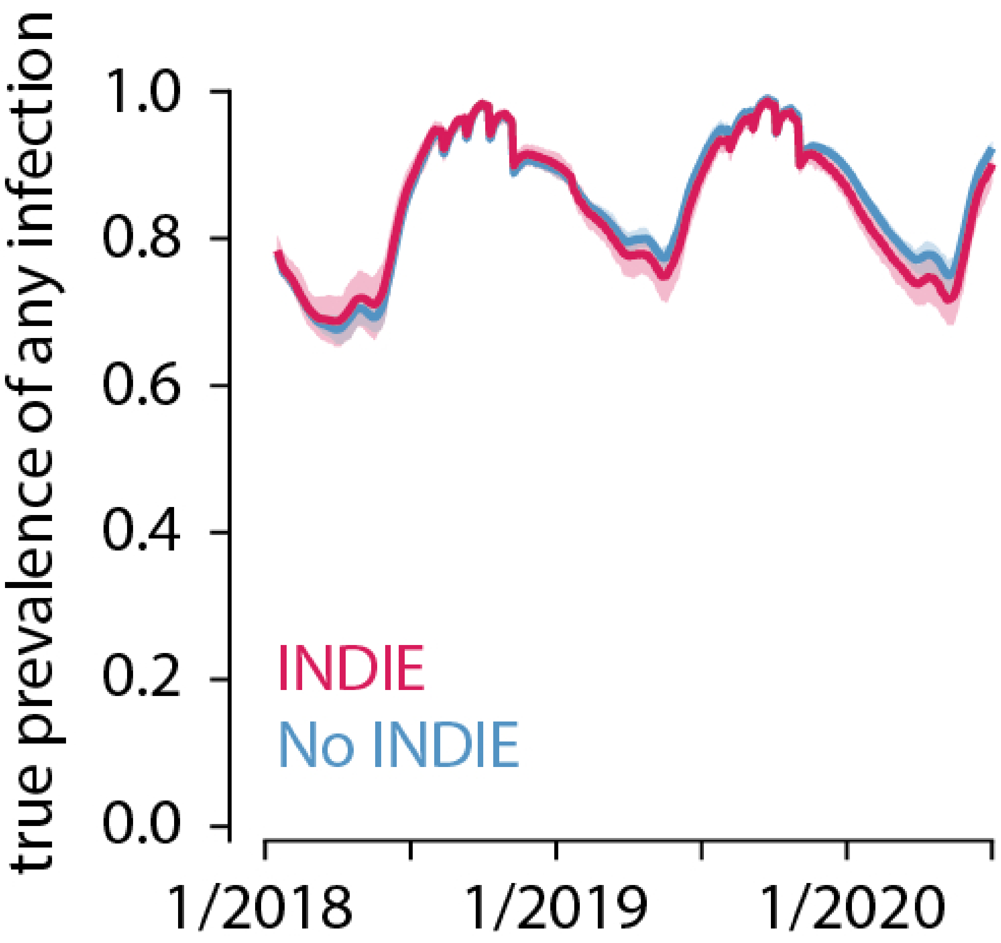
true prevalence of any infection
1.0
0.8
0.6
0.4
0.2
0.0
INDIE
No INDIE
1/2018
1/2019
1/2020

Figure 4

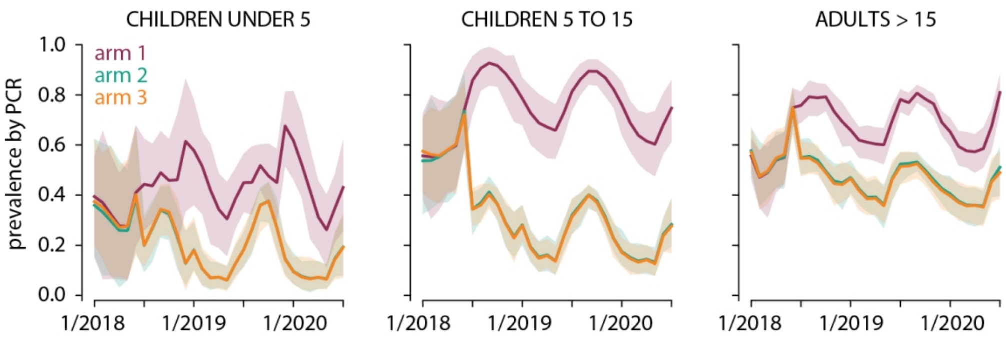
CHILDREN UNDER 5
CHILDREN 5 TO 15
ADULTS > 15
arm 1
arm 2
arm 3
prevalence by PCR
1.0
0.8
0.6
0.4
0.2
0.0
1/2018
1/2019
1/2020

Figure 5

Supplementary Information

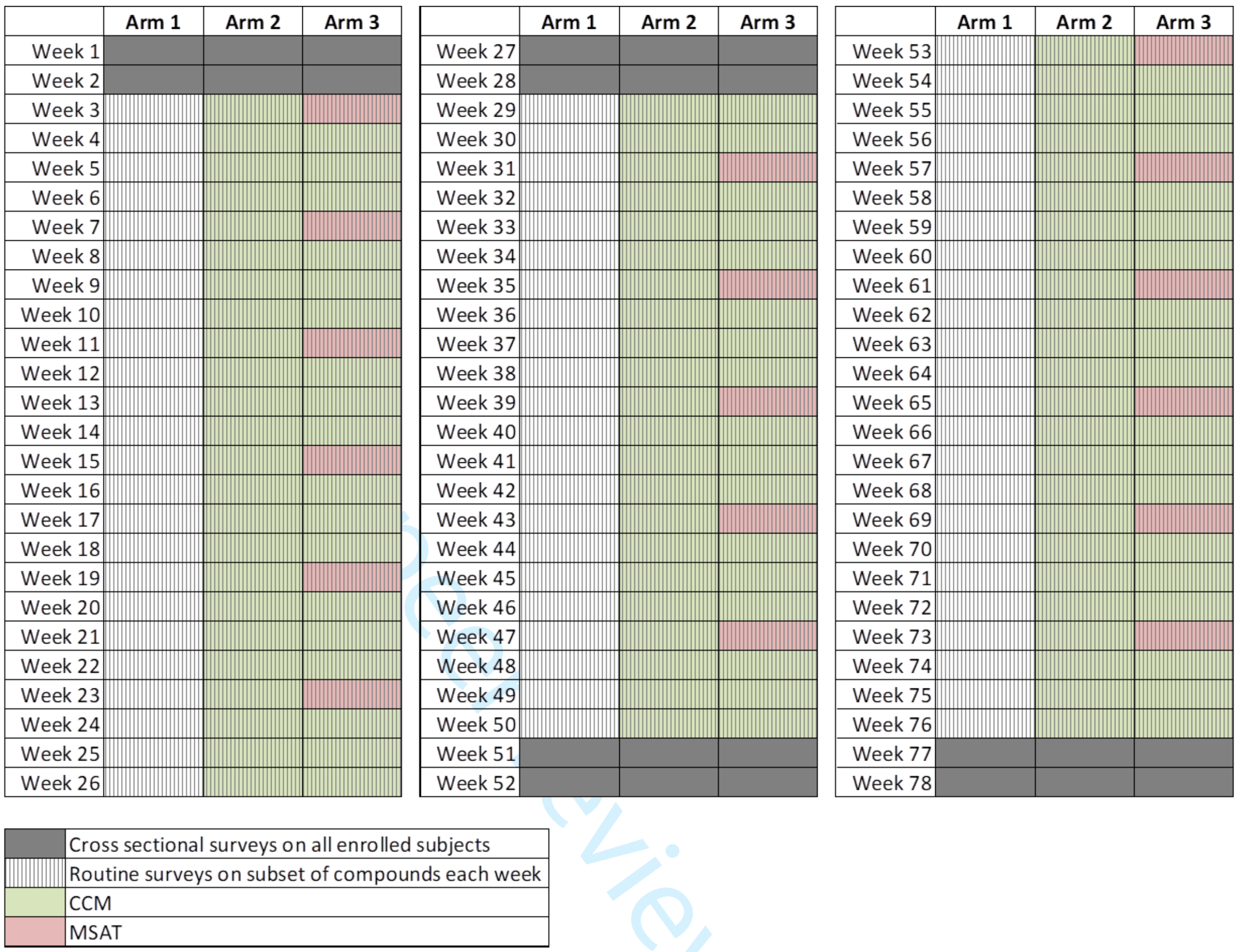

Figure S1. Timetable of trial activities

CCM = enhanced community case management; MSAT = monthly screening and treatment

For peer review only

Data category	Information
**Primary registry and trial identifying number**	ClinicalTrials.gov: NCT03705624
**Date of registration in primary registry**	July 19, 2018
**Secondary identifying numbers**	INDIE-1a
**Source(s) of monetary or material support**	The Bill and Melinda Gates Foundation
**Primary sponsor**	London School of Hygiene and Tropical Medicine<br>Keppel Street<br>London WC1E 7HT<br>Tel: +44 207 927 2626<br>Email: RGIO@lshtm.ac.uk
**Secondary sponsor(s)**	N/A
**Contact for public queries**	Teun Bousema, PhD +31 0243617574 (teun.bousema@radboudumc.nl)<br>Katharine Collins, PhD +31 0243613663 (katharine.collins@radboudumc.nl)<br>Chris Drakeley, PhD +44 02079272289 (chris.drakeley@lshtm.ac.uk)<br>Alfred Tiono, MD, PhD +226 50324695 (t.alfred@fasonet.bf)
**Contact for scientific queries**	TeunBousema, PhD +31 243617574 (teun.bousema@radboudumc.nl)<br>Chris Drakeley, PhD +44 2079272289 (chris.drakeley@lshtm.ac.uk)<br>Alfred Tiono, MD, PhD +226 50324695 (t.alfred@fasonet.bf)<br>Katharine Collins, PhD+31 243613663 (katharine.collins@radboudumc.nl)
**Public title**	P. falciparum Infection Dynamics and Transmission to Inform Elimination (INDIE 1a)
**Scientific title**	A cluster-randomized trial investigating the impact of enhanced community case management and monthly screening and treatment on the transmissibility of malaria infections in Burkina Faso
**Countries of recruitment**	Burkina Faso
**Health condition(s) or problem(s) studied**	Malaria
**Intervention(s)**	<u>Arm 1 (control): Standard of care</u><br>• Standard of care with passively monitored malaria incidence<br><u>Arm 2 (intervention): CCM</u><br>• Standard of care plus enhanced Community Case Management for Malaria (CCM) involving weekly active screening for fever. A measured temperature ≥37.5°C or reported fever in last 24h will prompt screening with a conventional rapid diagnostic test (RDT). RDT positive individuals will be treated with artemether-lumefantrine according to national guidelines<br><u>Arm 3 (intervention): CCM + MSAT</u><br>• Standard of care with enhanced CCM plus monthly screening and treatment regardless of symptoms using a conventional RDT (MSAT). RDT positive individuals will be treated with artemether-lumefantrine according to national guidelines.
**Key inclusion and exclusion criteria**	<u>Inclusion Criteria:</u><br>1. Participants should be permanent residents of the compound<br>2. Participants should be willing to participate in repeated assessments of health and infection status and willing to donate a maximum of 37mL of blood (children <10 years of age) or 52mL of blood (older individuals) during an 18-month period<br><br><u>Exclusion Criteria:</u><br>1. Any (chronic) illness that would affect with study participation<br>2. Pre-existing severe chronic health conditions

For peer review only - http://bmjopen.bmj.com/site/about/guidelines.xhtml

	3. Current participation in malaria vaccine trials or participation in such trials in the last 2 years<br>4. History of intolerance to artemether-lumefantrine
**Study type**	Interventional<br>Allocation: randomized<br>Intervention model: Cluster randomized trial<br>Masking: none (open label)<br>Primary purpose: Diagnostic
**Date of first enrolment**	June 2018
**Target sample size**	900
**Recruitment status**	Recruiting
**Primary outcome(s)**	Parasite prevalence and density between arms by molecular detection at the end of study cross-sectional survey [Time Frame: Month 18 (end of second transmission season; January-February 2020)].
**Key secondary outcomes**	1. Parasite prevalence and density by molecular detection at the end of year 1 cross-sectional survey [Time Frame: Month 6 (end of first transmission season; January-February 2019)].<br>2. Parasite prevalence and density by molecular detection at the end of the dry season cross-sectional survey [Time Frame: Month 12 (prior to second transmission season; June 2019)].<br>3. Gametocyte prevalence and or density by molecular methods at the end of study cross-sectional survey [Time Frame: Month 18 (end of second transmission season; January-February 2020)].<br>4. Gametocyte prevalence and or density by molecular methods at the end of year 1 cross-sectional survey [Time Frame: Month 6 (end of first transmission season; January-February 2019)].<br>5. Gametocyte prevalence and or density by molecular methods at the end of the dry season cross-sectional [Time Frame: Month 12 (prior to second transmission season; June 2019)].<br>6. Gametocyte prevalence and or density by molecular methods among *P. falciparum* infections during all visits in the study [Time Frame: Throughout study, an average of 18 months].<br>7. The number of incident infections/clinical incidence detected during CCM, MSAT and passive case detection [Time Frame: Throughout study, an average of 18 months].<br>8. Infectivity to mosquitoes of *P. falciparum* infections [Time Frame: Throughout study, an average of 18 months].

Table S1. World Health Organization Trial Registration Data Set

For peer review only

SPIRIT

STANDARD PROTOCOL ITEMS: RECOMMENDATIONS FOR INTERVENTIONAL TRIALS

SPIRIT 2013 Checklist: Recommended items to address in a clinical trial protocol and related documents*

Section/item	Item No	Description	Addressed on page number
**Administrative information**			
Title	1	Descriptive title identifying the study design, population, interventions, and, if applicable, trial acronym	___1__________
Trial registration	2a	Trial identifier and registry name. If not yet registered, name of intended registry	___2__________
	2b	All items from the World Health Organization Trial Registration Data Set	___S1+2______
Protocol version	3	Date and version identifier	___15_________
Funding	4	Sources and types of financial, material, and other support	___17________
Roles and responsibilities	5a	Names, affiliations, and roles of protocol contributors	___17________
	5b	Name and contact information for the trial sponsor	___S1________
	5c	Role of study sponsor and funders, if any, in study design; collection, management, analysis, and interpretation of data; writing of the report; and the decision to submit the report for publication, including whether they will have ultimate authority over any of these activities	___17________
	5d	Composition, roles, and responsibilities of the coordinating centre, steering committee, endpoint adjudication committee, data management team, and other individuals or groups overseeing the trial, if applicable (see Item 21a for data monitoring committee)	___16_____

For peer review only

Introduction

Background and rationale	6a	Description of research question and justification for undertaking the trial, including summary of relevant studies (published and unpublished) examining benefits and harms for each intervention	__4+5_______
	6b	Explanation for choice of comparators	__4+5________
Objectives	7	Specific objectives or hypotheses	__11+12______
Trial design	8	Description of trial design including type of trial (eg, parallel group, crossover, factorial, single group), allocation ratio, and framework (eg, superiority, equivalence, noninferiority, exploratory)	__6+7________

Methods: Participants, interventions, and outcomes

Study setting	9	Description of study settings (eg, community clinic, academic hospital) and list of countries where data will be collected. Reference to where list of study sites can be obtained	__7_________
Eligibility criteria	10	Inclusion and exclusion criteria for participants. If applicable, eligibility criteria for study centres and individuals who will perform the interventions (eg, surgeons, psychotherapists)	__7+8_________
Interventions	11a	Interventions for each group with sufficient detail to allow replication, including how and when they will be administered	__6, 8+9______
	11b	Criteria for discontinuing or modifying allocated interventions for a given trial participant (eg, drug dose change in response to harms, participant request, or improving/worsening disease)	__N/A________
	11c	Strategies to improve adherence to intervention protocols, and any procedures for monitoring adherence (eg, drug tablet return, laboratory tests)	__6, 15+16____
	11d	Relevant concomitant care and interventions that are permitted or prohibited during the trial	__6________
Outcomes	12	Primary, secondary, and other outcomes, including the specific measurement variable (eg, systolic blood pressure), analysis metric (eg, change from baseline, final value, time to event), method of aggregation (eg, median, proportion), and time point for each outcome. Explanation of the clinical relevance of chosen efficacy and harm outcomes is strongly recommended	__11+12________
Participant timeline	13	Time schedule of enrolment, interventions (including any run-ins and washouts), assessments, and visits for participants. A schematic diagram is highly recommended (see Figure)	___Figure 1___

For peer review only - http://bmjopen.bmj.com/site/about/guidelines.xhtml

Sample size	14	Estimated number of participants needed to achieve study objectives and how it was determined, including clinical and statistical assumptions supporting any sample size calculations	__13________
Recruitment	15	Strategies for achieving adequate participant enrolment to reach target sample size	__6__________

Methods: Assignment of interventions (for controlled trials)

Allocation:

Sequence generation	16a	Method of generating the allocation sequence (eg, computer-generated random numbers), and list of any factors for stratification. To reduce predictability of a random sequence, details of any planned restriction (eg, blocking) should be provided in a separate document that is unavailable to those who enrol participants or assign interventions	__7_________
Allocation concealment mechanism	16b	Mechanism of implementing the allocation sequence (eg, central telephone; sequentially numbered, opaque, sealed envelopes), describing any steps to conceal the sequence until interventions are assigned	__N/A_________
Implementation	16c	Who will generate the allocation sequence, who will enrol participants, and who will assign participants to interventions	__N/A_________
Blinding (masking)	17a	Who will be blinded after assignment to interventions (eg, trial participants, care providers, outcome assessors, data analysts), and how	__N/A_________
	17b	If blinded, circumstances under which unblinding is permissible, and procedure for revealing a participant's allocated intervention during the trial	__N/A_________

Methods: Data collection, management, and analysis

Data collection methods	18a	Plans for assessment and collection of outcome, baseline, and other trial data, including any related processes to promote data quality (eg, duplicate measurements, training of assessors) and a description of study instruments (eg, questionnaires, laboratory tests) along with their reliability and validity, if known. Reference to where data collection forms can be found, if not in the protocol	___8+9_______
	18b	Plans to promote participant retention and complete follow-up, including list of any outcome data to be collected for participants who discontinue or deviate from intervention protocols	___15_______

Data management	19	Plans for data entry, coding, security, and storage, including any related processes to promote data quality (eg, double data entry; range checks for data values). Reference to where details of data management procedures can be found, if not in the protocol	___15+16_______
Statistical methods	20a	Statistical methods for analysing primary and secondary outcomes. Reference to where other details of the statistical analysis plan can be found, if not in the protocol	___13_____
	20b	Methods for any additional analyses (eg, subgroup and adjusted analyses)	___13________
	20c	Definition of analysis population relating to protocol non-adherence (eg, as randomised analysis), and any statistical methods to handle missing data (eg, multiple imputation)	___N/A________
**Methods: Monitoring**			
Data monitoring	21a	Composition of data monitoring committee (DMC); summary of its role and reporting structure; statement of whether it is independent from the sponsor and competing interests; and reference to where further details about its charter can be found, if not in the protocol. Alternatively, an explanation of why a DMC is not needed	___16______
	21b	Description of any interim analyses and stopping guidelines, including who will have access to these interim results and make the final decision to terminate the trial	___7________
Harms	22	Plans for collecting, assessing, reporting, and managing solicited and spontaneously reported adverse events and other unintended effects of trial interventions or trial conduct	___15+16_______
Auditing	23	Frequency and procedures for auditing trial conduct, if any, and whether the process will be independent from investigators and the sponsor	___15+16_______
**Ethics and dissemination**			
Research ethics approval	24	Plans for seeking research ethics committee/institutional review board (REC/IRB) approval	___15_______
Protocol amendments	25	Plans for communicating important protocol modifications (eg, changes to eligibility criteria, outcomes, analyses) to relevant parties (eg, investigators, REC/IRBs, trial participants, trial registries, journals, regulators)	___15________

Consent or assent	26a	Who will obtain informed consent or assent from potential trial participants or authorised surrogates, and how (see Item 32)	__15_______
	26b	Additional consent provisions for collection and use of participant data and biological specimens in ancillary studies, if applicable	__N/A________
Confidentiality	27	How personal information about potential and enrolled participants will be collected, shared, and maintained in order to protect confidentiality before, during, and after the trial	__15________
Declaration of interests	28	Financial and other competing interests for principal investigators for the overall trial and each study site	__17________
Access to data	29	Statement of who will have access to the final trial dataset, and disclosure of contractual agreements that limit such access for investigators	__N/A________
Ancillary and post-trial care	30	Provisions, if any, for ancillary and post-trial care, and for compensation to those who suffer harm from trial participation	__15_______
Dissemination policy	31a	Plans for investigators and sponsor to communicate trial results to participants, healthcare professionals, the public, and other relevant groups (eg, via publication, reporting in results databases, or other data sharing arrangements), including any publication restrictions	__16_________
	31b	Authorship eligibility guidelines and any intended use of professional writers	__N/A________
	31c	Plans, if any, for granting public access to the full protocol, participant-level dataset, and statistical code	__16________
**Appendices**			
Informed consent materials	32	Model consent form and other related documentation given to participants and authorised surrogates	__N/A________
Biological specimens	33	Plans for collection, laboratory evaluation, and storage of biological specimens for genetic or molecular analysis in the current trial and for future use in ancillary studies, if applicable	__N/A________

*It is strongly recommended that this checklist be read in conjunction with the SPIRIT 2013 Explanation & Elaboration for important clarification on the items. Amendments to the protocol should be tracked and dated. The SPIRIT checklist is copyrighted by the SPIRIT Group under the Creative Commons "Attribution-NonCommercial-NoDerivs 3.0 Unported" license.
